# Characterization of fatty acid metabolism-related lncRNAs in lung adenocarcinoma identifying potential novel prognostic targets

**DOI:** 10.3389/fgene.2022.990153

**Published:** 2022-09-27

**Authors:** Yang Liu, Xingshu Zhang, Xuechao Cheng, Qian Luo, Mingyang Yu, Kaijun Long, Wendong Qu, Yang Tang, Ming Gong, Lubiao Liang, Xixian Ke, Yongxiang Song

**Affiliations:** Department of Thoracic Surgery, The Affiliated Hospital of Zunyi Medical University, Zunyi, Guizhou, China

**Keywords:** lung adenocarcinoma, long non-coding RNA, biomarker, fatty acid metabolism, immune cells

## Abstract

Lung adenocarcinoma (LUAD), a malignant respiratory tumor with an extremely poor prognosis, has troubled the medical community all over the world. According to recent studies, fatty acid metabolism (FAM) and long non-coding RNAs (lncRNAs) regulation have shown exciting results in tumor therapy. In this study, the original LUAD patient data was obtained from the TCGA database, and 12 FAM-related lncRNAs (AL390755.1, AC105020.6, TMPO-AS1, AC016737.2, AC127070.2, LINC01281, AL589986.2, GAS6-DT, AC078993.1, LINC02198, AC007032.1, and AL021026.1) that were highly related to the progression of LUAD were finally identified through bioinformatics analysis, and a risk score model for clinical reference was constructed. The window explores the immunology and molecular mechanism of LUAD, aiming to shed the hoping light on LUAD treatment.

## Introduction

Lung cancer accounts for the largest share of cancer-related deaths worldwide (Thai et al., 2021). It is worth noting that lung adenocarcinoma (LUAD) accounts for up to 85% of lung cancers and is the most common subspecies (Pao and Girard, 2011) (Nicholson et al., 2021). Based on different molecular and pathological features, LUAD can be subdivided into various subtypes (Inamura, 2018). There are differences and connections between different subtypes, but the commonalities between them are high malignancy, poor prognosis, and greater difficulty in early diagnosis (Blandin Knight et al., 2017) (Rami-Porta et al., 2018). From the perspective of treatment, the current treatment methods for LUAD mainly include surgery (Zappa and Mousa, 2016), immunotherapy (Hellmann et al., 2018), targeted therapy (Arbour and Riely, 2017), etc. However, various treatment methods are limited by the histology of LUAD, mutated genes, and differences in clinical stages, and the prognosis of patients is often not exactly (Zappa and Mousa, 2016). Coupled with the low sensitivity of LUAD to radiotherapy (Zappa and Mousa, 2016) and the gradual emergence of resistance to targeted therapy drugs (Remon et al., 2021), we are forced to have a deeper understanding of LUAD.

Tumor cells are often in an abnormal metabolic environment, depending on the imbalance between the rapid proliferation of tumor cells and nutrient angiogenesis (Yi et al., 2018; Han et al., 2021). Modern thinking holds that tumor cells need to reprogram their metabolism to meet increased metabolic and synthetic demands in conjunction with their own growth needs, while simultaneously reducing the negative effects of oxidative stress during growth (Martínez-Reyes and Chandel, 2021). Collectively, tumor metabolic reprogramming is significant (Hanahan and Weinberg, 2011), and the change of aerobic glycolysis (Warburg effect), glutamine metabolism, and one-carbon *de novo* synthesis of fatty acids also confer the ability of tumors to rapidly progress in a relatively nutrient-stressed tumor microenvironment (TME) (Cluntun et al., 2017; Newman and Maddocks, 2017; Ashton et al., 2018; Zhao et al., 2020; Li et al., 2021a). Interestingly, changes in the metabolic level of tumor cells often lead to changes in the components of the TME, thereby having a significant impact on affecting the biological effects of other cellular components of the TME, and these changes will ultimately affect tumor progression (Dey et al., 2021) (Broadfield et al., 2021). It is worth noting that fatty acid metabolism (FAM), as one of the important pathways of the three major nutrients metabolism, can be coupled with a variety of metabolic pathways and participate in cell membrane formation, intracellular signal transduction, hormone secretion, and other processes, and is related with the disease and health state in human (Kimura et al., 2020) (Bogie et al., 2020). At the same time, the relationship between FAM and cancer progression has received increasing attention (Koundouros and Poulogiannis, 2020) (Bergers and Fendt, 2021). long non-coding RNAs (lncRNAs) are a class of RNAs with regulatory functions, and they have been extensively studied in the past decade (Ali and Grote, 2020). Previous studies have illustrated that lncRNAs are vital in cell cycle regulation (Jiang et al., 2021), metabolic regulation (Tan et al., 2021), and even the immune system (Fok et al., 2018), and have been recognized as playing a significant role in cancer progression (Wu et al., 2020).

Here, we constructed a 12 FAM related-lncRNAs signature risk model based on LUAD raw data in TCGA by bioinformatic methods. Further immunological and functional analysis indicated the possible mechanism of action of these lncRNAs in LUAD and their impact on the first immunotherapy of LUAD. And at the end of the study, polymerase chain reaction (PCR) technology was conducted to verify the expression of the screened lncRNAs.

## Materials and methods

### Data preparation and processing

All kinds of LUAD data were obtained from the TCGA database (http://portal.gdc.cancer.gov/) (Blum et al., 2018). With previous reports about FAM-related genes and Kyoto Encyclopedia of Genes and Genomes (KEGG) databases (Kanehisa et al., 2017), 1879 FAM-related lncRNAs were obtained by using the correlation test between the FAM-related genes and lncRNAs with R. The thresholds were set as |cor|>0.4 and *p* < 0.001. LUAD patients without overall survival (OS) values or whose OS was within 30 days were excluded. Four hundred and ninety samples were divided into training and testing sets randomly. 246 samples were contained in the training set, while contained 244 in the testing set.

### Establishment and validation of the risk signature

With survival information, we screened the prognosis of FAM-related lncRNAs from 1879 differently expressed lncRNAs (*p* < 0.05). Univariate Cox regression analysis was used to screen lncRNAs related to survival. LASSO regression was performed by R package “glmnet” (version 4.1-3) with 10-fold cross-validation, 1,000 cycles. With the Multifactor Cox regression, a 12 FAM-related lncRNA risk model was finally built.

The risk score was calculated by the following formula:

$$Risk\;score=\sum_{k=1}^{n}Coef\left(\ln cRNA\right)*\exp\left(\ln cRNA^{k}\right)$$

where Coef is the coefficient and exp is the expression level of lncRNA.

The mean score was regarded as a standard to distinguish LUAD subgroups.

### Model performance estimation

The univariate and multivariate Cox (by “glment,” “survminer,” and “survival” R packages) regression analyses were developed to evaluate the independent predictive power of risk models. The 1-,3-, and 5-year ROC curves were used to evaluate the effect of prognostic prediction. Principal component analysis (PCA) and t-distributed Stochastic Neighbor Embedding (t-SNE) analysis were further used to verify the risk model.

### Nomogram and calibration

A nomogram was established based on our risk score model and various clinical characteristics by the “rms” R package. The 1-, 3-, and 5-year OS and ROC curves were performed to illustrate the actual consistency of the model with the practical.

### The investigation of the TME and immunotherapy

The mutation data was the sum and analyzed by R package maftools. The infiltration status of immune cells, TME scores, and immune checkpoints activation between two different subgroups were presented *via* CIBERSORT and ssGSEA algorithm and visualized by the “ggpubr” R package. The Tumor Immune Dysfunction and Exclusion (TIDE) algorithm was also used to predict the likelihood of the immunotherapeutic response, Immunotherapeutic treatment data from the website (http://tide.dfci.harvard.edu/) (Wang et al., 2020). The data of the immune subtype was downloaded on TIMER (http://timer.comp-genomics.org/) (Li et al., 2017).

### Exploration of the model in the clinical treatment

The R package “pRRophetic” was used to evaluate the therapy response of each LUAD patient on Genomics of Drug Sensitivity in Cancer (GDSC) (Yang et al., 2013). Drug sensitivity analyses are conducted online (https://discover.nci.nih.gov/cellminer/home.do).

### Functional analysis

Differentially expressed genes (DEGs) between two groups were identified by using the package “limma” following the criteria (|Log2FC| > 1.0, *p*-value < 0.05). GO and KEGG enrichment analysis was applied using the package “clusterProfiler” in R. GSEA analysis was conducted to further screen functional pathways by using software GSEA 4.2.1 (http://www.gesa-msigdb.org/gsea/index,jsp) (Powers et al., 2018). Furthermore, the competitive endogenous RNA (ceRNA) network between lncRNAs and mRNAs was visualized by Cytoscape (version 3.6.1).

### RNA extraction and real-time quantitative PCR

We extracted total RNA from the samples. We synthesized cDNA using a ServicebioRT First Strand cDNA Synthesis Kit (Applied Servicebio, China). Then, cDNA was subjected to a Real-Time Quantitative Polymerase Chain Reaction (RT-qPCR) by the bio-rad CFX (Applied Bio-rad, China). We used b-actin mRNA as an internal reference to normalize the nine lncRNAs by the comparative Ct method. All three cell lines (H1299, A549, and BEAS-2B) were purchased from Procell. The ambient temperature was controlled at 37°C and the CO_2_ concentration was 5%. The three cell lines were added to a 1640 medium containing 10% fetal bovine serum and incubated in a constant temperature incubator.

### Statistical analysis

All statistical analyses were conducted in the R software (Version 4.1.1). Wilcoxon rank-sum test was used to compare the difference between the two groups. K-W test was performed to compare three or more groups. Kaplan-Meier analysis was used to evaluate the survival differences between the low- and high-risk score groups.

If there is no special description for the above method, statistical significance is defined as a *p*-value < 0.05.

## Results

### FAM-related lncRNAs in LUAD patients

**FIGURE 1:**
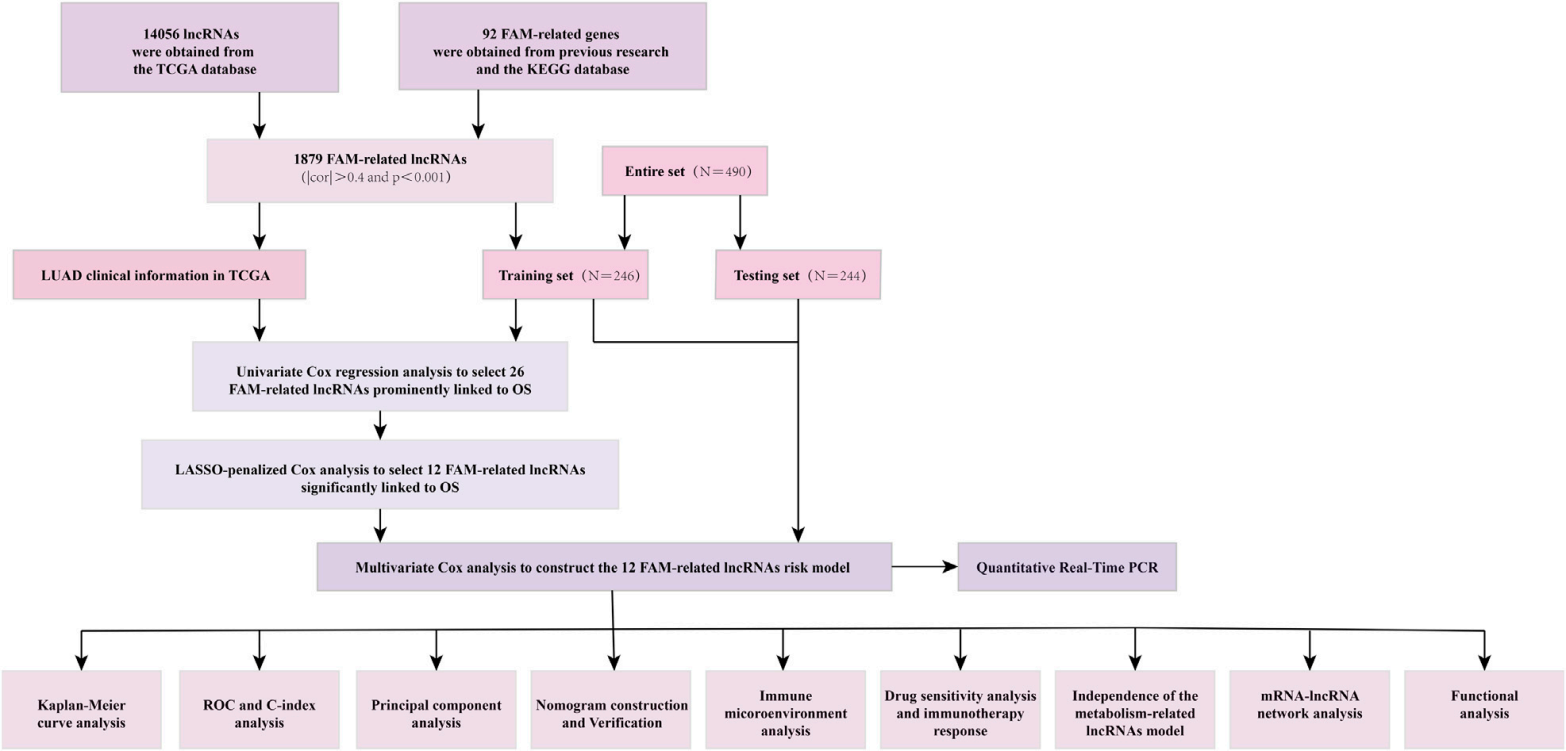
The entire analytical process of the study.

**FIGURE 2:**
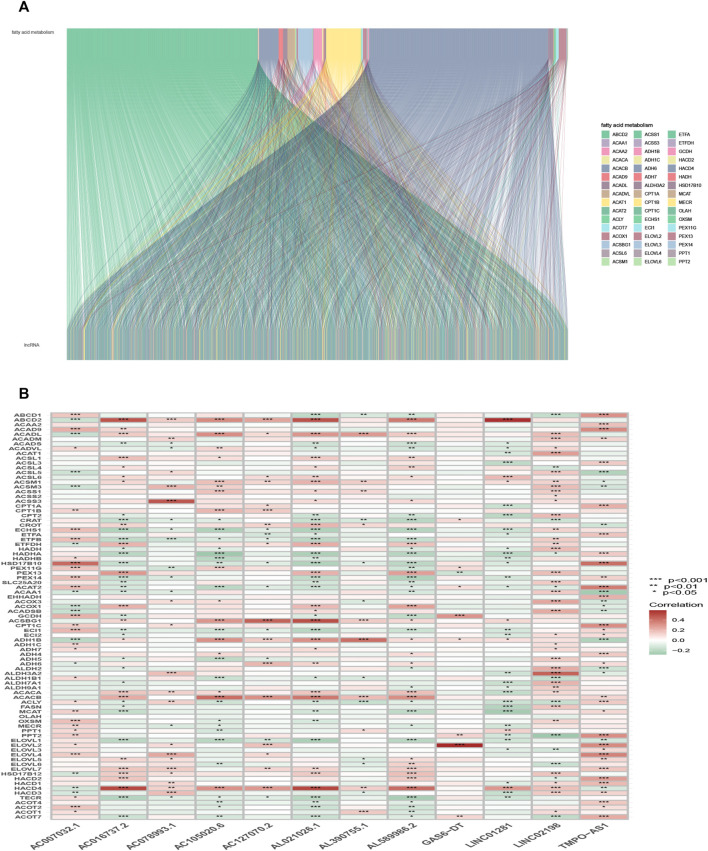
Selection of FAM-related lncRNAs in LUAD patients. **(A)** Sankey relation diagram for target lncRNAs. **(B)** Heatmap of the correlation between FAM-related genes and the 12 prognostic FAM-related lncRNAs in TCGA entire set.

The detailed process is shown in Figure 1. A total of 490 LUAD patients were included in this analysis, with their clinical features in Table 1. 92 FAM-related genes (Appendix D1) were obtained from previous research and the KEGG database. By using Pearson correlation analysis, 1879 FAM-related lncRNAs were discerned as FAM lncRNAs (Figure 2A). The relationship data between FAM-related genes and lncRNAs were shown in Appendix D2, and their correlation was shown in (Figure 2B) (Part of fatty acids metabolism-related genes were selected for display and Supplementary Figure S1 for all).

**TABLE 1:** The clinical characteristics of included samples.

Covariates	Type	Total	Test	Train	*p*-value
Age	≤65	231 (47.14%)	111 (45.49%)	120 (48.78%)	0.5789
	>65	249 (50.82%)	127 (52.05%)	122 (49.59%)	
	unknown	10 (2.04%)	6 (2.46%)	4 (1.63%)	
Gender	FEMALE	262 (53.47%)	136 (55.74%)	126 (51.22%)	0.3618
	MALE	228 (46.53%)	108 (44.26%)	120 (48.78%)	
Stage	Stage I-II	378 (77.14%)	184 (37.55%)	194 (39.59%)	0.8546
	Stage III-IV	69 (21.22%)	55 (11.22%)	49 (10%)	
	unknown	8 (1.63%)	5 (2.05%)	3 (1.22%)	
T	T1-2	426 (86.94%)	210 (42.86%)	216 (44.08%)	0.7023
	T3-4	61 (12.45%)	32 (6.53%)	29 (5.92%)	
	unknown	3 (0.61%)	2 (0.82%)	1 (0.41%)	
M	M0	324 (66.12%)	165 (67.62%)	159 (64.63%)	0.7872
	M1	24 (4.9%)	11 (4.51%)	13 (5.28%)	
	unknown	142 (28.98%)	68 (27.87%)	74 (30.08%)	
N	N0	317 (64.69%)	157 (64.34%)	160 (65.04%)	0.3002
	N1-3	162 (33.06%)	82 (16.73%)	80 (16.33%)	
	unknown	11 (2.24%)	5 (2.05%)	6 (2.44%)	

### Construction and validation of a prognostic model

**FIGURE 3:**
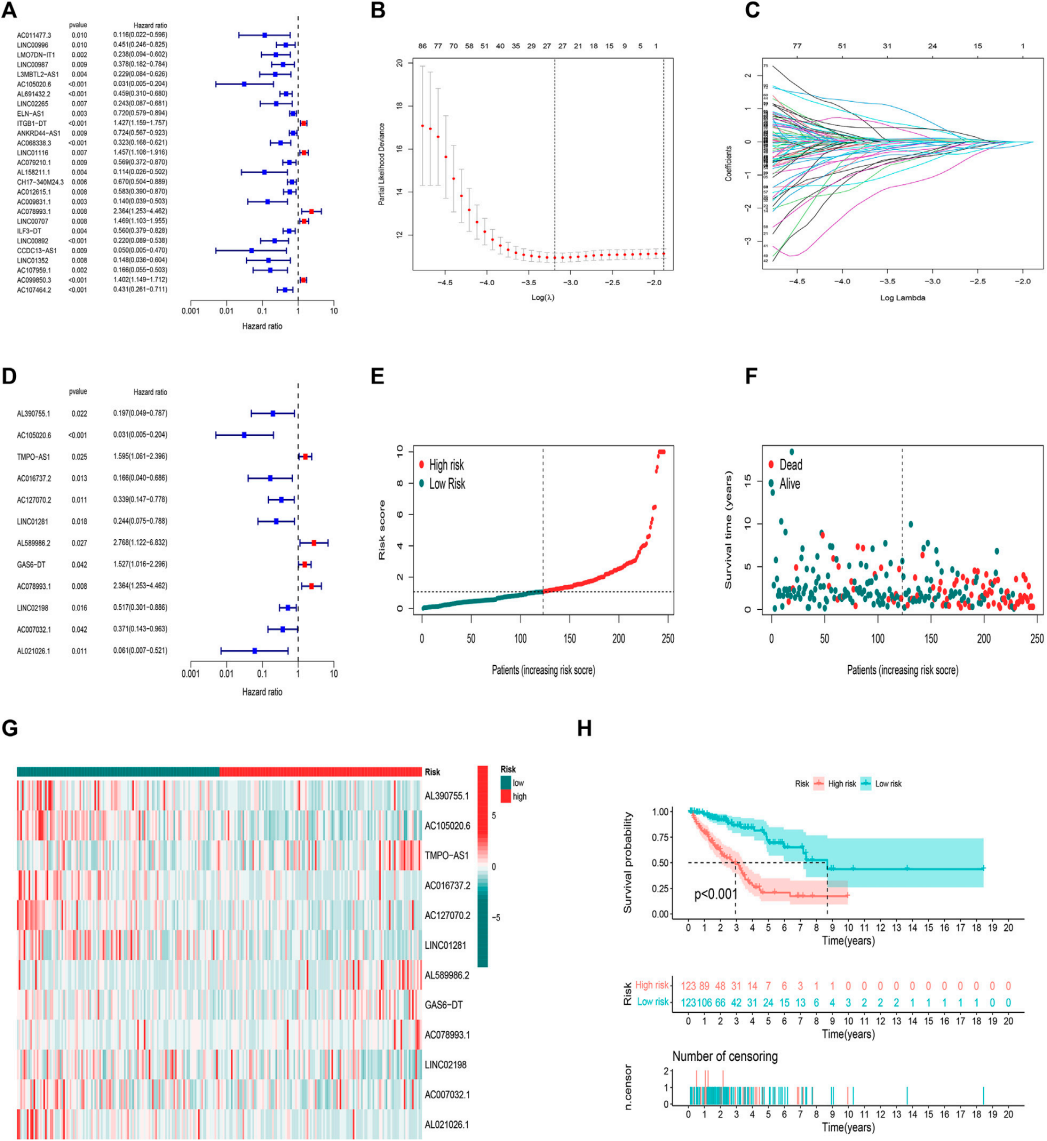
Prognostic model in training set validation. **(A)** Univariate Cox regression analysis. **(B)** The LASSO coefficient profile. **(C)** The 10-fold cross-validation for variable selection in the LASSO model. **(D)** Multivariate Cox regression analysis and 12 lncRNAs were finally selected. **(E)** Patient risk score distribution for the training set. **(F)** Survival status time between two risk groups in the training set. **(G)** 12 FAM-related lncRNAs distributed for each patient in the training set. **(H)** OS curve of the training set.

Here, 164 FAM-related lncRNAs were identified through univariate COX regression analysis (Figure 3A, results whose *p* < 0.01 were selected to show and all results were available in Supplementary Figure S2. The LASSO regression focused on 26 related lncRNAs while avoiding overfitting (Figures 3B,C). Finally, 12 FAM-related lncRNAs (Table 2) were used to construct this prognostic model (Figure 3D).

**TABLE 2:** The 12 FAM-related prognostic lncRNAs.\

Id	Coef	HR	HR.95L	HR.95H	*p*-value
AL390755.1	−1.548137522	0.196552779	0.049061185	0.787445217	0.021594278
AC105020.6	−2.549155904	0.031057225	0.004725325	0.204123802	0.000301471
TMPO-AS1	0.645364341	1.594883853	1.061457403	2.396379259	0.024635498
AC016737.2	−1.625290281	0.165930576	0.040164005	0.685513212	0.013077694
AC127070.2	−1.129022783	0.338535109	0.147220182	0.778466772	0.010790501
LINC01281	−1.219080278	0.243707993	0.075401385	0.787698871	0.018341205
AL589986.2	1.976381633	2.768161634	1.121511954	6.832489666	0.027193217
GAS6-DT	1.048808092	1.527058692	1.015808552	2.295617855	0.041813182
AC078993.1	0.651117829	2.36441459	1.252924785	4.461924947	0.007910449
LINC02198	−0.520620804	0.516815504	0.301364474	0.886296453	0.016458722
AC007032.1	−1.431274742	0.371231383	0.143127389	0.9628677	0.041572162
AL021026.1	−1.552575457	0.060826538	0.007094814	0.521489027	0.010653806

The risk score was evaluated as: risk score = AL390755.1×(−1.54813752178063)+ AC105020.6×(−2.54915590358082)+ TMPO-AS1×(0.645364340734862)+ AC016737.2×(−1.62529028103815)+ AC127070.2×(−1.1290227830944)+ LINC01281×(−1.21908027803503)+ AL589986.2×(1.97638163310763)+ GAS6-DT×(1.04880809216376)+ AC078993.1×(0.651117829305934)+ LINC02198×(−0.520620804335338)+ AC007032.1×(−1.43127474226429)+ AL021026.1×(−1.55257545730318)

The median value of the risk score was the standard to divide LUAD samples. All samples were divided into two low-/high-risk groups. The distribution of risk grades and survival information between the two groups is shown in (Figures 3E,F). The relative expression standards of the 12 FAM-related lncRNAs for each patient are shown in (Figure 3G). The survival analysis demonstrated that the OS of the low-risk group was longer than that of the high-risk group (Figure 3H *p* < 0.001).

**FIGURE 4:**
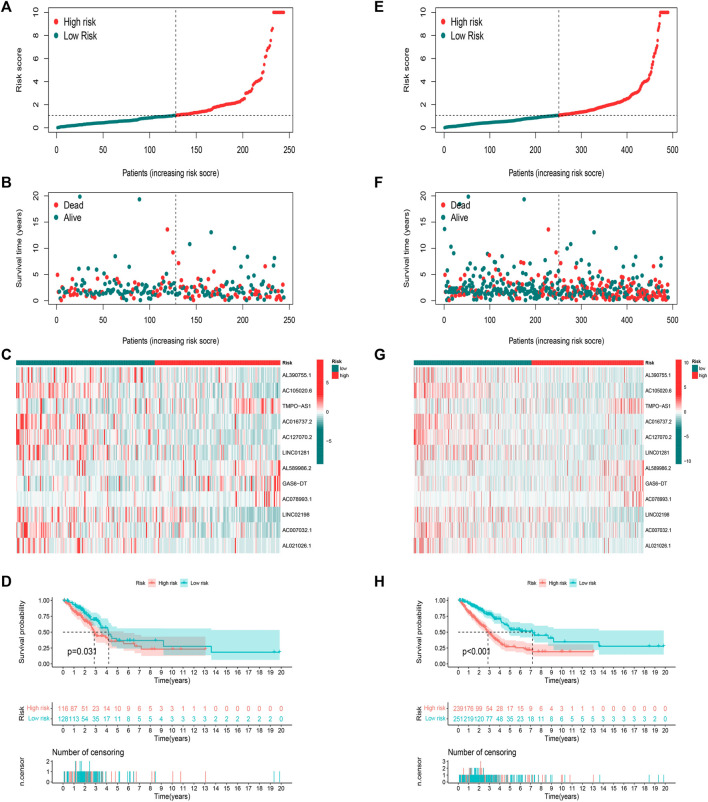
Prognostic value of risk score model in testing and entire sets. **(A**–**D)** Distribution of risk score, survival status, 12 hub lncRNA expression levels, and K-M survival curve (OS) in the testing set. **(E**–**H)** Distribution of risk score, survival status, 12 hub lncRNA expression levels, and K-M survival curve (OS) in the entire set.

We calculated risk scores for LUAD patients to validate the predictive capability of the established model by using the uniform formula. Figure 4 shows the diffusion of risk scores, survival status and time, and expression of the FAM-related lncRNAs in the testing set (Figures 4A–C) and the entire set (Figures 4E–G). The K-M survival curve based on the testing set and the entire set also showed that the patients in the low-risk group had a longer OS than those in the high-risk group (Figures 4D,H *p* < 0.05).

### PCA

**FIGURE 5:**
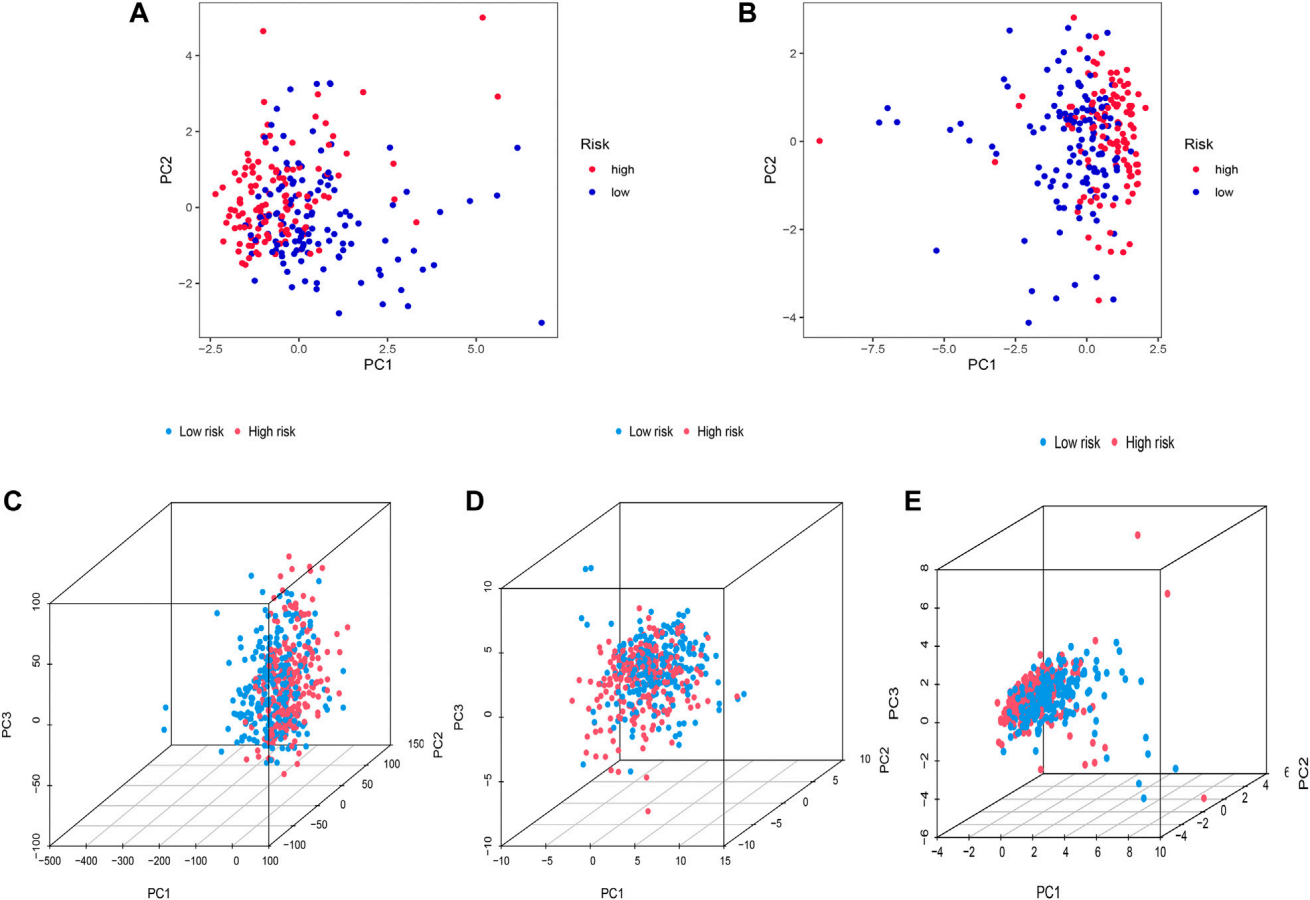
Principal component analysis. **(A**,**B)** 2D PCA in training and the entire set. **(C**–**E)** PCA between two risk groups for entire gene expression profiles, 92 FAM related-genes, and profiles of the 12 FAM-related lncRNAs as an entire set.

Heterogeneity between the two risk subgroups in the entry set and test set was examined by PCA analysis. The whole gene expression profiles, 92 FAM genes, as well as our risk model was included (Figure 5). The analysis results according to the risk model we constructed showed that the low- and high-risk groups had different distributions (Figures 5C–E). This shows that the risk model can distinguish between low- and high-risk groups.

### Nomogram

**FIGURE 6:**
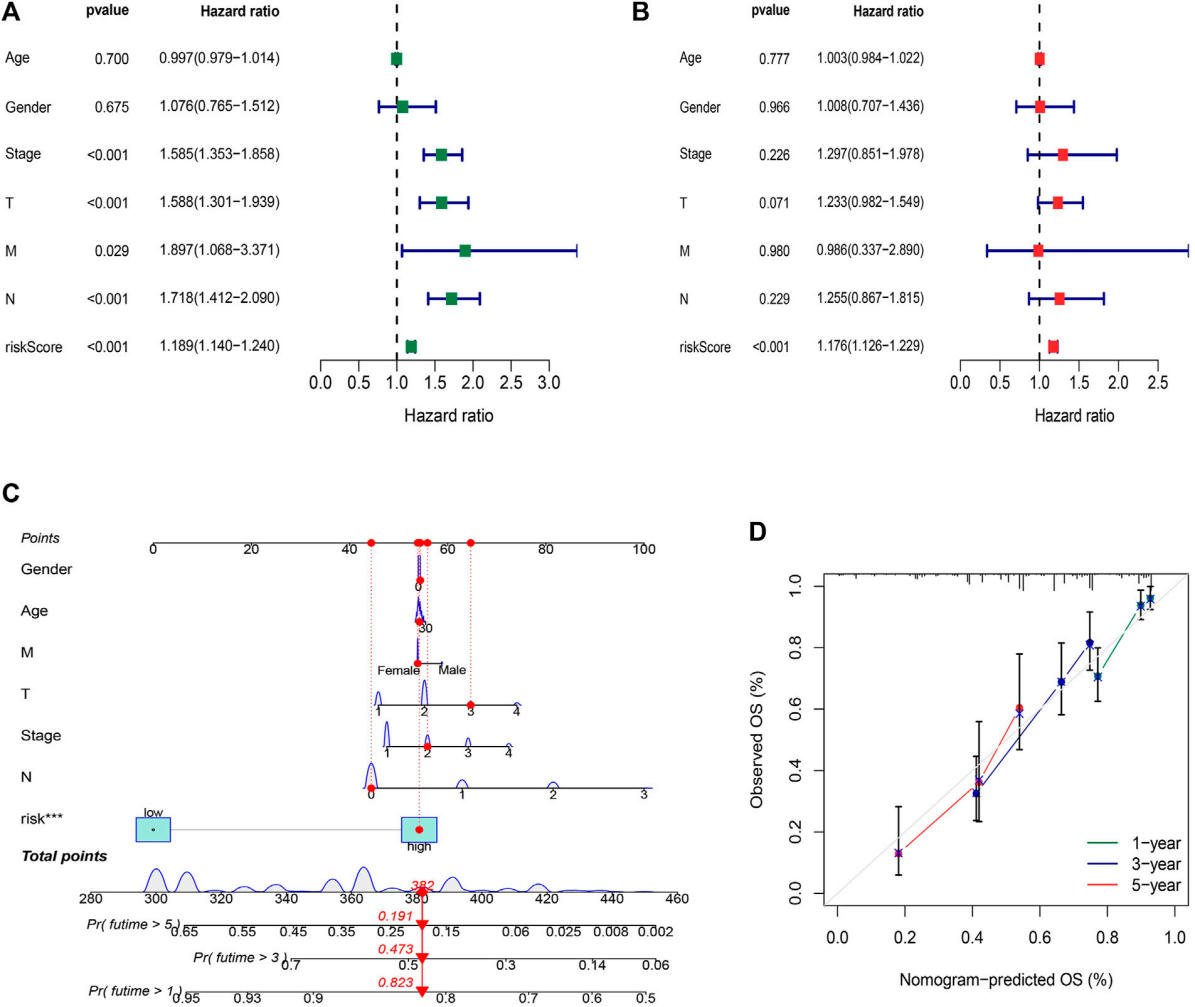
Construction and validation of the nomogram. **(A)** Univariate Cox regression analysis indicated that disease stage, T stage, M stage, and risk score, were related to prognosis (*p* < 0.001) **(B)** Multivariate Cox regression analysis presented that the risk score was an independent factor affecting prognosis (*p* < 0.001). **(C)** The nomogram predicts the probability of the 1-, 3-, and 5-year OS. **(D)** The calibration plot.

The hazard ratio (HR) of the risk score and 95% confidence interval (CI) were 1.189 and 1.140–1.240 (*p* < 0.001), respectively, in univariate Cox (uni-Cox) regression while 1.176 and 1.126–1.229 (*p* < 0.001), respectively, in multivariate Cox (multi-Cox) regression (Figures 6A,B). Univariate Cox regression analysis indicated that disease stage, T stage, M stage, and risk score, were related to prognosis (Figure 6A, *p* < 0.001). Furthermore, multivariate Cox regression analysis presented that the risk score was an independent factor affecting prognosis (Figure 6B, *p* < 0.001). Therefore, we are reasonably confident that risk models based on FAM-related lncRNAs have a significant impact on the survival and prognosis of LUAD patients and are independent prognostic factors. To better predict the 1-,3-, and 5-year survival for LUAD patients, we established a nomogram combining gender, age, stage, TNM, and risk score (Figure 6C). Using calibration curve analysis, the prediction accuracy of the nomogram was assessed (Figure 6D).

### Assessment of the risk model

**FIGURE 7:**
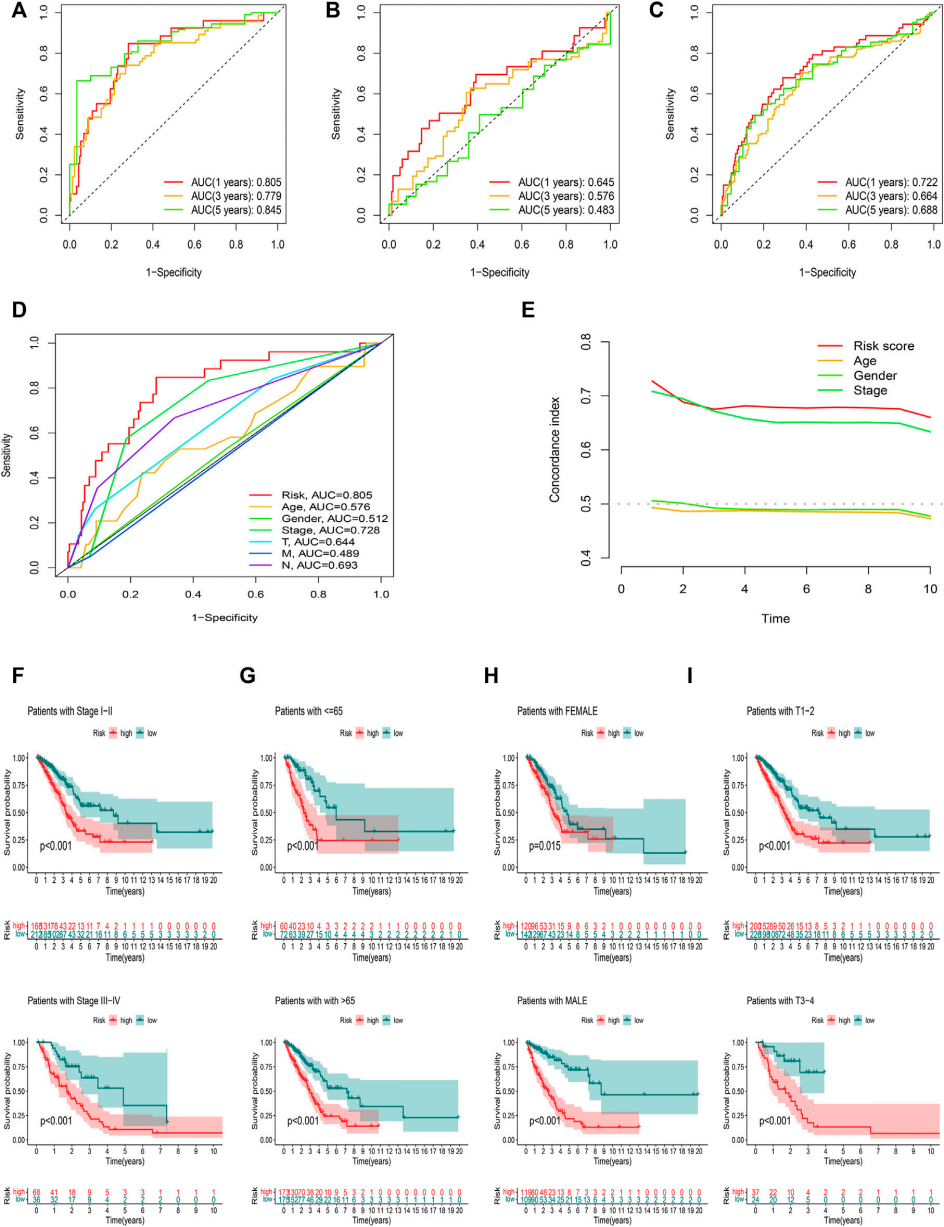
Assessment of the prognostic risk model. **(A**–**C)** The 1-, 3-, and 5-year ROC curves of the training, testing set, and entire set. **(D)** ROC curves of all included features. **(E)** CI of the risk score and clinical characteristics. **(F**–**I)** OS curve of difference clustered by LUAD clinical features between two risk groups in the entire set.

ROC curves were utilized to evaluate the sensitivity and specificity of the model on the prognosis. The AUC (1-, 3-, and 5-year) for the train set were 0.805, 0.779, and 0.845, of the test set were 0.645, 0.576, and 0.483, and of the entire set were 0.722, 0.664, and 0.688, respectively (Figures 7A–C). The AUC value illustrated that the prognostic risk model of the 12 FAM-related lncRNAs for LUAD was comparatively dependable (Figures 7D,E).

Figures 7F–I showed the OS of patients after sub-clustering using clinical characteristics based on the risk score. Like the previous results, the OS of the low-risk group was better than that of the high-risk group.

### Stratification analysis of the risk model in immune features

**FIGURE 8:**
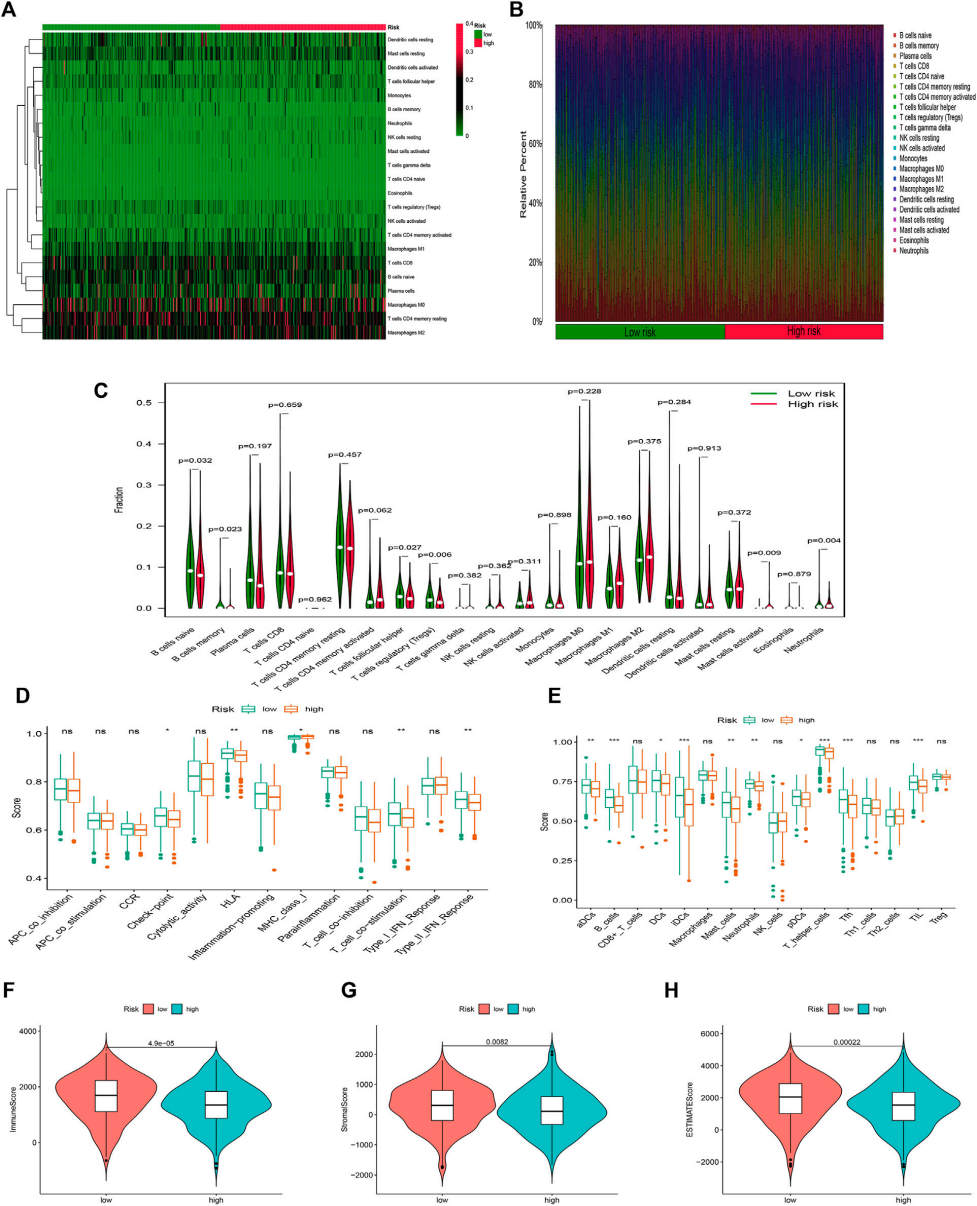
Stratification Analysis of the FAM-related lncRNA prognostic risk score in immune features. **(A**–**C)** Heatmap, bar chart, and relative infiltrating proportion of 22 tumor-infiltrating immune cell types in two risk groups. **(D**,**E)** The score of immune functions comparing two risk groups by ssGSEA or ssGSEA score. **(F**–**H)** The comparison of immune-related scores between high- and low-risk groups.

The infiltration status of immune cells was evaluated by the CIBERSORT algorithm. The proportions of 22 immune cells in each sample were shown in Figures 8A,B. The high-risk group was associated with significantly lower levels of B cells, T cells follicular helper, and Tregs, but a higher level of eosinophils and neutrophils (Figure 8C). Subsequently, the results of the ssGSEA algorithm showed that the high-risk group had a lower mean infiltration level than the low-risk group, with T helper cells showing higher infiltration levels in both risk groups (Figures 8D,E). We obtained similar results in correlation analysis of immune responses, and overall, patients in the high-risk group had a lower immune response. In addition, LUAD patients in the high-risk group had remarkably lower stromal, immune, and ESTIMATE scores (Figures 8F–H).

**FIGURE 9:**
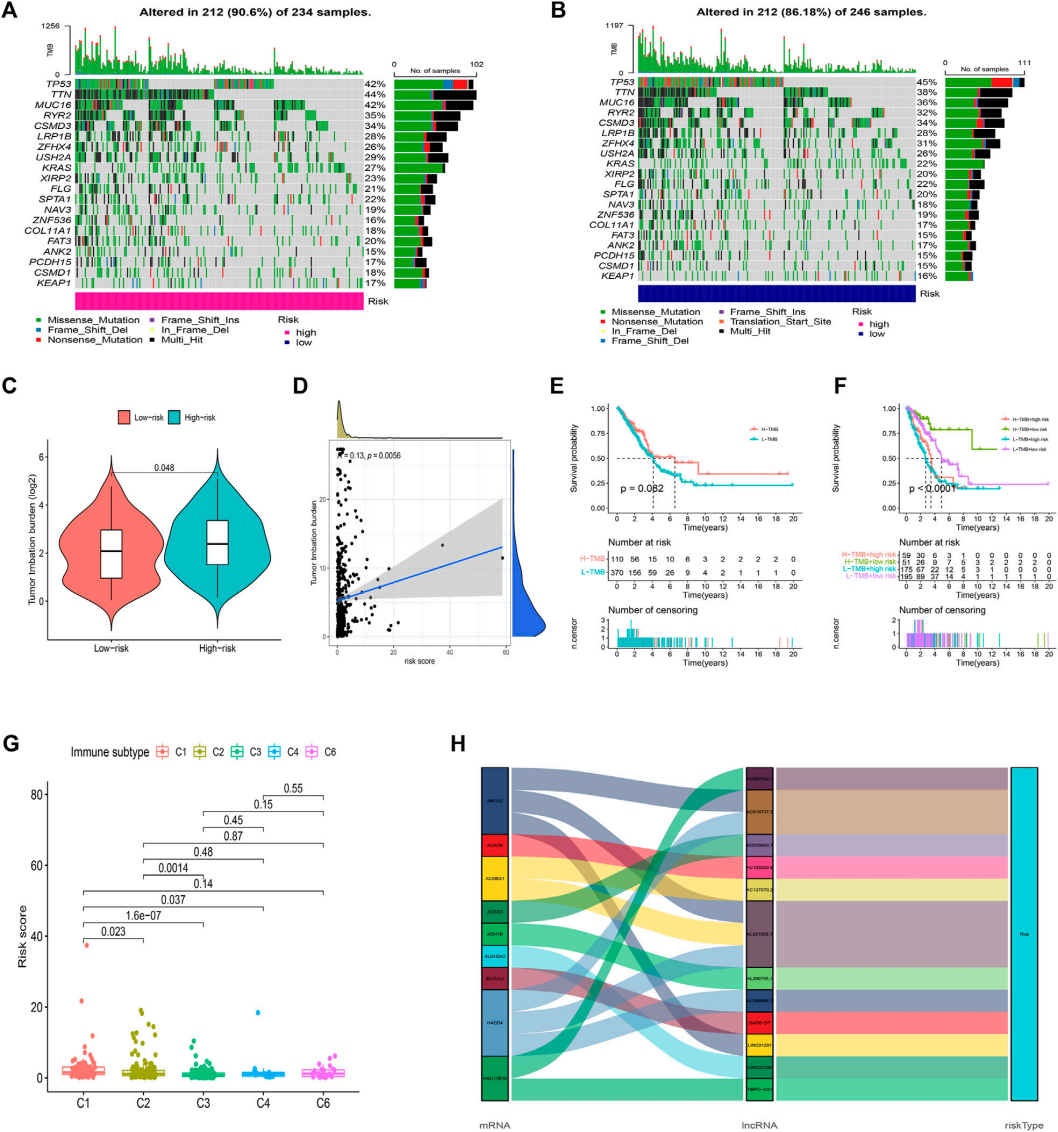
Exploration of TMB and lncRNAs networks visualization. **(A**,**B)** 20 genes with high mutation frequencies in different risk subgroups. **(C)** TMB difference in two risk groups. **(D)** The correlation between risk score and TMB. **(E)** K-M curves of the patient OS in the high-TMB and low-TMB groups in the entire set. **(F)** The survival outcome predictive validity of TMB. **(G)** The correlation between risk score and immune subtype. **(H)** Sankey diagram: the connection degree between the FAM-related genes, FAM-related lncRNAs, and risk types.

We then analyzed the mutation data. Mutations were stratified according to the constructed risk model. The results of mutations analysis with those top 20 driver genes are shown in Figures 9A,B. A higher level of TP53 mutations was correlated with a worse survival state. The TMB in the high-risk group exceeded that in the low-risk group, showing that the FAM-related risk model classifier index had a high correlation with TMB (Figure 9C). Therefore, we tested the correlation between FAM-related lncRNAs and TMB based on the risk model using Spearman correlation analysis (Figure 9D r = 0.13, *p* = 0.0056). The results suggested a strong correlation between the FAM-based classifier index and the TMB. We further investigated the impact of TMB status on the prognosis of LUAD patients by analyzing the survival of the high and low TMB groups. However, the survival curves were similar in both groups, indicating that TMB failed to differentiate survival in LUAD (Figure 9E, *p* > 0.05). Besides, the survival outcome (OS) predictive validity of TMB was conducted, which shows a weaker predictive power than our risk model (Figure 9F, *p* < 0.05). The results show that our model may predict better than the TMB.

Furthermore, according to TIMER2.0 data (Appendix D3), we divided all samples into different immune subtypes (Figure 9G). FAM genes, 12 FAM-related lncRNAs, and risk types were included in the Sankey network (Figure 9H). The above results illustrate the high correlation of these 12 FAM-related lncRNAs with LUAD immunity from another dimension.

### Clinical treatment and drug sensitivity analysis

**FIGURE 10:**
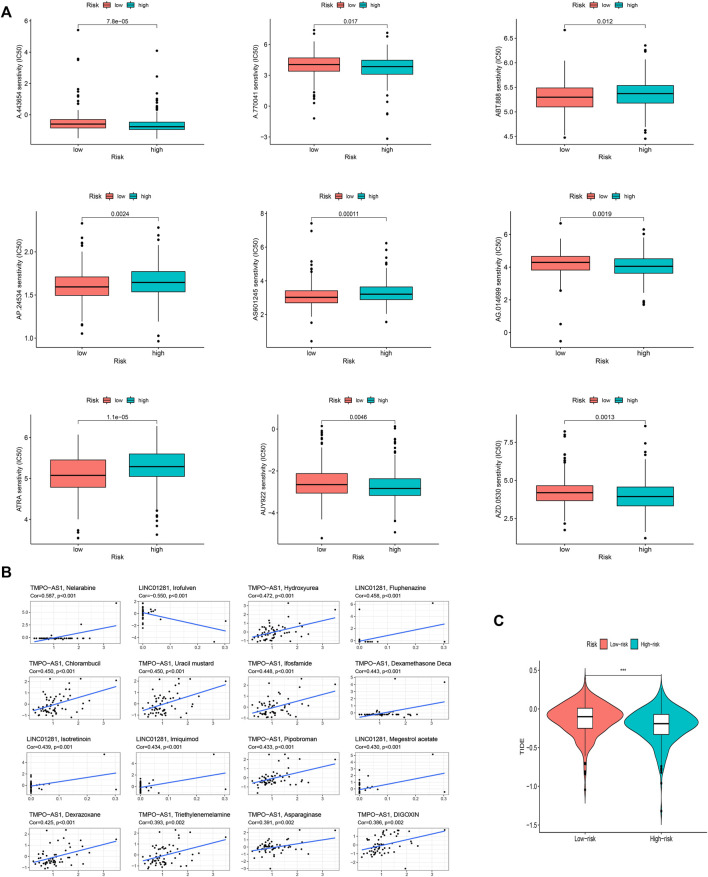
The investigation of tumor immune factors and immunotherapy. **(A)** The immunotherapy prediction of high-risk and low-risk groups. **(B)** The correlation between 12 FAM-related lncRNAs and drugs. **(C)** TIDE prediction difference in the high-risk and low-risk patients.

Given the differences in the immune microenvironment between these two risk groups, we hypothesized that these two groups might have different responses to drugs. We then used the pRophetic algorithm to estimate treatment response against potential drugs in our model based on the IC50 of each sample in the GDSC database. The correlation between CI50 and different risk groups were shown in Figure 10A. The IC50s for AP.24534, ATRA, AS601245, and ABT.888 were significantly higher in the low-risk group (Figure 10A), suggesting that exposure to these drugs may be more appropriate for high-risk patients. Then those model-related lncRNAs and immunotherapeutic biomarkers were pooled to explore their relationship. We were pleasantly surprised to find that TMPO-AS1 was related to the sensitivity of multiple drugs (Figure 10B, the entire result was available in Appendix D4).

Unsurprisingly, the high-risk group may effect better in immunotherapy, which also means that our model might serve as a potential signature for predicting TIDE (Figure 10C).

### Functional analysis

**FIGURE 11:**
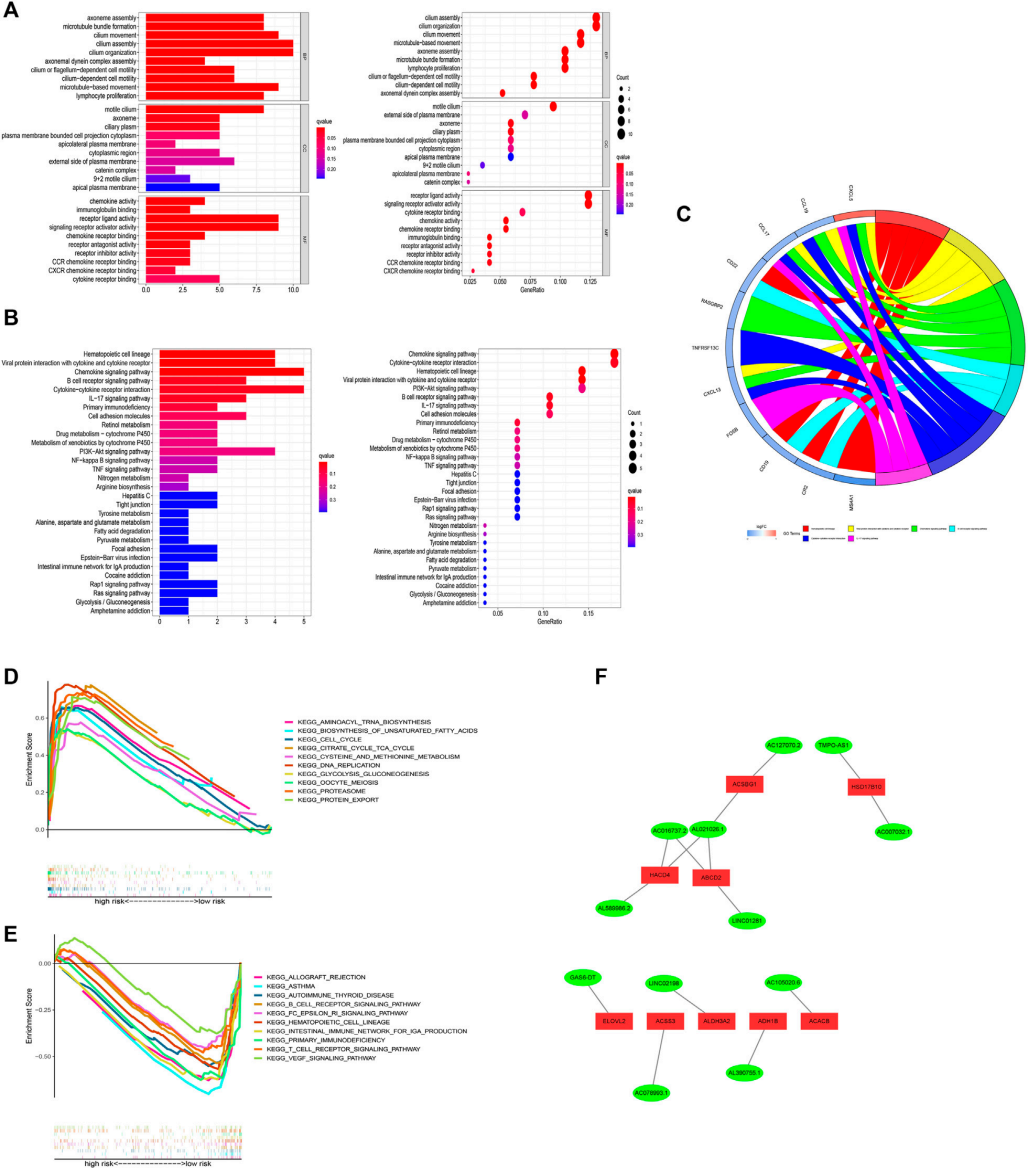
Functional analysis. **(A)** Result of GO functional enrichment (top 10). **(B)** KEGG enrichment terms (top 30). **(C)** Circle diagram in KEGG analysis. **(D)** GSEA of the top 10 pathways significantly enriched in the high-risk group. **(E)** GSEA of the top 10 pathways in the low-risk group. **(F)** 12 FAM-related lncRNAs and differential FAM genes networks.

GO analysis illustrated that these risk model-related genes mainly affect the modulation of axoneme assembly, motile cilium, chemokine activity, and so on (Figure 11A). KEGG analysis illustrated that these genes were involved in multiple immune pathways such as the chemokine signaling pathway, B cell receptor signaling pathway, and so on (Figures 11B,C). KEGG analysis results were shown in Figures 11D,E. Pathways such as aminoacyl tRNA biosynthesis and biosynthesis of unsaturated fatty acids were significantly related to the high-risk group, while pathways such as allograft rejection and asthma were significantly enriched in the low-risk group. In addition, we also established an interaction network for these key lncRNAs (Figure 11F).

### Verification of expression level *in vitro* on hub lncRNAs

**FIGURE 12:**
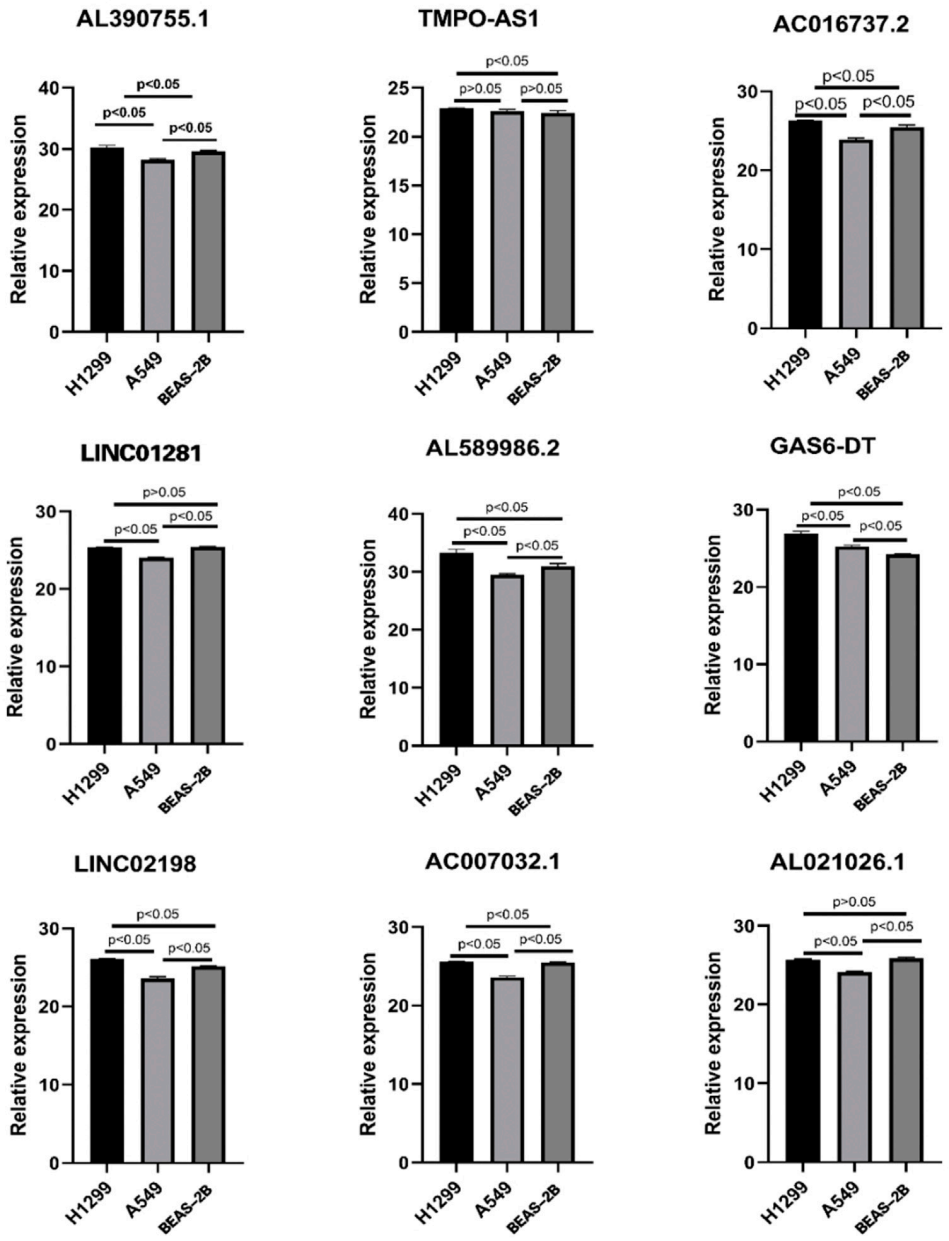
Expression of nine lncRNAs from the risk model in LUAD cell lines and bronchial epithelial cells.

To verify the expression level of 12 FAM-related lncRNAs in LUAD cells, we used RT-qPCR analysis to detect BEAS-2B and LUAD cells, including A549 and H1299 (Figure 12). Unfortunately, the sequences of three lncRNAs (AC105020.6, AC127070.2, AC078993.1) did not have suitable primers, so we only verified the expression levels of the remaining nine FAM-related lncRNAs (The PCR primer sequences were available in Table 3). Among these lncRNAs, we found that the expression of GAS6-DT in the A549 and H1299 cell lines was significantly higher than that in the BEAS-2B cell line, and TMPO-AS1 was significantly higher in the H1299 cell line. Combined with the previous research results, their high expression was associated with a poorer prognosis, with HR < 1, suggesting that GAS6-DT and TMPO-AS1 genes may be a risk factor in LUAD. The expression levels of AL021026.1 and LINC01281 in the A549 cell line were significantly lower than those in the BEAS-2B cell line, and their average expression in the H1299 cell line was lower than that in BEAS-2B, but the difference was not significant (*p* > 0.05). Model coefficients and patient outcomes are considered that they may be protective factors for LUAD. Interestingly, we also noticed that LINC02198, AC007032.1, AL589986.2, and AL390755.1 were significantly overexpressed in the H1299 cell line and significantly underexpressed in the A549 cell line, while AC016737.2 was significantly overexpressed in H1299 cell line. The high expression contradicts the result of its coefficient of less than 0 in the risk model.

**TABLE 3:** The PCR primer sequences.

Gene	F 5′-3′	R 5′-3′
TMPO-AS1	5ʹ-CAG​ACC​TCT​ACA​ATC​GGG​CAC​TTA-3′	5ʹ-ATT​CTT​GCG​GGT​GGT​GGG​AT-3′
AC016737.2	5ʹ-CTG​GAG​ATG​GAC​TTT​GGC​T-3′	5ʹ-CTT​GTG​AGG​TGG​CTG​TTA​TTA​TC-3′
LINC01281	5ʹ-CAG​CCC​AGA​GTG​AAG​ATA​AGA​ATA​C-3′	5ʹ-GAA​GCC​ACC​AGC​AGA​ATG​ACA-3′
GAS6-DT	5ʹ-TAG​CTA​TTA​TTT​CCT​AAG​GGT​TCC​AG-3′	5ʹ-TCC​ATT​AAC​TCT​CTT​CTC​CAA​AAC​TAC​A-3′
LINC02198	5ʹ-ACT​TCT​GTC​ACC​CCC​TTG​ATT​ACC-3′	5ʹ-CCA​AAG​ACT​GGT​CCT​CCT​CTA​TCC-3′
AC007032.1	5ʹ-TGA​TGA​CTT​CAC​CCA​AAT​ACA​GAC​C-3′	5ʹ-ACT​TTT​TCC​TGG​CTA​CTT​TTA​TCC​G-3′
AL021026.1	5ʹ-ATA​TCT​GAG​CCT​GAG​TTT​CCC​ATT​C-3′	5ʹ-TTC​CAT​AGC​CGC​CAA​TAC​AAG​C-3′
AL390755.1	5ʹ-GGA​AAG​CTA​TGA​GGA​AGA​AGA​AAC​AGA-3′	5ʹ-CAA​CCT​GTG​CTG​TGA​TGA​ATG​G-3′
AL589986.2	5ʹ-CCT​GAT​ACT​GGT​TTT​TCT​ACA​TGC​TTC-3′	5ʹ-TCC​AAG​GTT​GTG​CTA​TGG​TAA​TCT​G-3′

## Discussion

With the deepening of tumor research, the role of metabolic reprogramming in tumors cannot be underestimated any longer (Faubert et al., 2020). Simply, tumor cells are different from normal tissue cells, when tumor cells are ready to colonize other organs, they need to compete with other normal cells for the living environment and nutrients. Therefore, the metabolic demands of tumor cells are regulated to meet the needs of survival in the current environment (Schild et al., 2018). Based on the above characteristics, the metabolic reprogramming of tumor cells is also regarded as a hallmark of tumor development (Ward and Thompson, 2012). In addition, more and more evidence shows that in addition to protein-coding RNA mutations, mutations and abnormal modifications of non-coding RNAs represented by lncRNAs were also vital in tumor progression (Bhan et al., 2017). Therefore, these non-coding RNAs also play a key role in tumor progression. It is regarded as a new marker for tumor diagnosis or a new therapeutic target (Kong et al., 2019; Wang et al., 2019; Xing et al., 2021). Here, we cannot help but want to explore whether and how lncRNA can interact with the lipid metabolism reprogramming of tumor cells, and how the interaction between the two affects the process of LUAD and thus affects the prognosis and survival of patients. At the same time, it is hoped that more powerful biomarkers and therapeutic targets can be found for the clinical diagnosis and treatment of LUAD.

In this study, LUAD data were obtained from the TCGA database, while FAM-related lncRNA data were downloaded from the KEGG database. After differential gene analysis, 1879 differential lncRNAs related to FAM were found, and after survival analysis, univariate/multifactor and LASSO Cox regression. A 12-hub FAM-related lncRNA prognostic model with high reliability and validity was constructed. Further exploration was performed to figure out how those hub lncRNAs were involved in LUAD progression.

Among the 12 key FAM-related genes we finally screened for risk score modeling, most lncRNAs have not been studied, but some lncRNAs have also appeared in the construction of prognosis prediction models for different diseases. For example, AL390755.1 was used to construct a prognostic prediction model for low-grade glioblastoma, and similar LINC01281, AL589986.2, and AC007032.1 were also used for laryngeal cancer (Zhang et al., 2019), cervical cancer (Ye et al., 2021), dilated cardiomyopathy (Zhang et al., 2020) and proliferative vitreoretinopathy respectively (Ni et al., 2021), which were also considered a potential diagnostic marker. At the same time, we also noticed that GAS6-DT and TMPO-AS1 have been shown to have regulatory axes in previous studies, which can interact with another coding/non-coding RNAs, and these two lncRNAs were proved to be possible risk factors for LUAD in our PCR validation. For example, the study of Zilin Li et al. pointed out that in liver cancer cells with incomplete radiofrequency ablation, the expression of GAS6-DT is often up-regulated and can competitively inhibit the binding of microRNA-3619-5p to ARL2, thereby promoting the proliferation and migration of liver cancer cells (Li et al., 2021b). The relevant research on TMPO-AS1 is relatively sufficient. The study of Xiaoqian Mu et al. (Mu et al., 2020) pointed out that TMPO-AS1 is highly expressed in LUAD samples and knocking down this gene negatively regulates the cell cycle of tumors and reduces the invasiveness of tumors. Targeted binding to TMPO-AS1 plays a role similar to gene knockout. A similar study by Qiu L et al. (Li et al., 2021c) also pointed out that TMPO-AS1 can also interact with miR-143-3p, ultimately affecting the expression of CDK1 and regulating the cell cycle of LUAD. Interestingly, the study by Jie Yao et al. (Yao et al., 2021) pointed out that TMPO-AS1 is also involved in the regulation of iron metabolism in LUAD.

When conducting drug sensitivity analysis, we were pleasantly surprised to find that the expression level of TMPO-AS1 is highly correlated with the sensitivity of various drugs, including ifosfamide, thiotepa, irinotecan, and other antitumor drugs that have been approved for clinical LUAD use. Among them, the expression level of this lncRNA is highly positively correlated with the CI50 of ifosfamide, which means that the higher the expression level of TMPO-AS1 (which also means a higher risk score), the worse the effect of ifosfamide for LUAD treatment. At the same time, we also noticed that trametinib showed a negative correlation. The experimental study by Toshiyuki Sumi et al. indicated that trametinib can reduce survivin expression in RB1^+^/KRAS-mutated LUAD cells, thereby improving prognosis (Sumi et al., 2018), and there was a similar case study by Maurício Fernando Silva Almeida Ribeiro et al. (Ribeiro et al., 2021). However, the latest clinical study by Luo J, Makhnin A et al. pointed out that in the drug-resistant EGFR-mutant LUAD that had previously appeared with a tyrosine kinase inhibitor, the addition of trametinib could not reverse the sensitivity of the tumor to the drug (Luo et al., 2021), which means that TMPO-AS1 may be a potential target to solve this problem, which is worthy of further study.

In addition, in the PCR validation of these hub lncRNAs, we found that the expression levels of the four lncRNAs LINC02198, AC007032.1, AL589986.2, and AL390755.1 were not consistent in the H1299 and A549 cell lines, and they were all expressed in the H1299 cell line. Moderately high expression, but low expression in the A549 cell line. We speculate that this may be related to the differences in the genomes of the two cells themselves. H1299 is a lymph node-derived human NSCLC cell line (Giaccone et al., 1992), while A549 cells are human adenocarcinoma alveolar basal epithelial cells (Foster et al., 1998). The different sources of the two may be one of the possibilities leading to this contradiction. Secondly, the H1299 cell line is considered to be a p53 wild-type cell, while the A549 is a p53-null cell (Dorandish et al., 2021). Several studies have also pointed to heterogeneity between the two types of cells, and our findings may add some new evidence to this topic (Yang et al., 2018) (Sidorova and Petrikaitė, 2022). In addition, we also noticed that AC016737.2 was significantly highly expressed in the H1299 cell line, but the coefficient of this lncRNA in the risk model was negative. This contradiction may require more basic experiments to explain.

Furthermore, with the deepening of research, there is a lot of evidence that there is a close relationship between the metabolic reprogramming of tumor cells and the tumor immune response (Cronin et al., 2018). In a broad sense, lipid metabolites include phospholipids, fatty acids, and cholesterol, and the impact of FAM on immune cells is particularly well-studied, for example, during the transformation of monocytes like neutrophils, the demand for fatty acid synthesis is significantly increased (den Hartigh et al., 2010). In regulatory T cells (Tregs), there is high fatty acid oxidation for energy, but in effector T cells, this oxidative activity is inhibited, which also maintains the relative stability of the immune system (Amersfoort et al., 2021). When we jointly analyzed the immune characteristics of LUAD samples of different risk groups, we found that the immune characteristics of the high-risk group were all suppressed compared with the low-risk group. At the level of immune cell infiltration, T helper cells (CD4^+^ T cells) were infiltrated to a higher degree in both high and low-risk groups, and there were significant differences between groups. Previous studies have pointed out that FAM is closely related to the phenotypic differentiation of T helper cells (Almeida et al., 2016), but our study found that Th1/Th2 subtypes did not have significant infiltration differences between the two risk groups, so we guessed that the lipid metabolism of LUAD is important. Programming may have more effect on the shift of T helper cells towards Th17. Recent work by Panagiota Mamareli indicated that *de novo* synthesis of fatty acids is necessary for the differentiation of the Th17 phenotype (Mamareli et al., 2021), In contrast, Ran You et al. observed that TIL in early-stage NSCLC was biased towards IL17A expression, whereas Th17 cells were reduced in tumor-infiltrating regional lymph nodes in advanced NSCLC (You et al., 2018). The clinical study by Chen G et al. also showed that Th17 and IL-17 increased in the peripheral blood of LUAD patients (Chen et al., 2020). Interestingly, in the functional enrichment analysis, we found that the IL-17 signaling pathway was enriched to the top position, which confirmed our conjecture to a certain extent. All the above evidence suggested that lipid metabolism reprogramming in LUAD may lead to the differentiation of T helper cells inclined towards Th17, which affects the LUAD process.

Interestingly, in the functional enrichment analysis, we found that the IL-17 signaling pathway was enriched to the top position, which confirmed our conjecture to a certain extent.

Overall, based on the lncRNA regulation of lipid metabolism in LUAD, we constructed a prognostic prediction model with good prediction results, and the model has high reliability and validity. In addition, we also conducted a preliminary study on how these key lncRNAs participate in the LUAD process and affect LUAD immunotherapy. Everything we do aims to improve the understanding of LUAD and shed a hoping light on early clinical diagnosis and treatment of LUAD.

### Limitations

Of course, almost all studies face certain challenges of limitations. Our study is no exception. First, we need to complete further validation experiments to provide reliable support for this study. Second, traditional statistical analysis methods may be of limited value in building and assessing prognostic risk models. We hope to open the door for lung adenocarcinoma research, and more questions to follow will require more investigators to join the study.

## Data Availability

The original contributions presented in the study are included in the article/Supplementary Material, further inquiries can be directed to the corresponding authors.

## Abbreviations

ceRNA, competitive endogenous RNA; FAM, fatty acid metabolism; GDSC, Genomics of Drug Sensitivity in Cancer; KEGG, Kyoto Encyclopedia of Genes and Genomes; lncRNAs, long non-coding RNAs; LUAD, Lung adenocarcinoma; OS, overall survival; PCA, principal component analysis; PCR, Polymerase Chain Reaction; RT-qPCR, Real-Time Quantitative Polymerase Chain Reaction; TIDE, Tumor Immune Dysfunction and Exclusion; TME, tumor microenvironment; t-SNE, t-distributed Stochastic Neighbor Embedding.
