# Supplementary figures and images for: Characterization of fatty acid metabolism-related lncRNAs in lung adenocarcinoma identifying potential novel prognostic targets
Source: Front Genet. 2022 Sep 27;13:990153. doi: 10.3389/fgene.2022.990153 (PMC9589892; doi:10.3389/fgene.2022.990153)

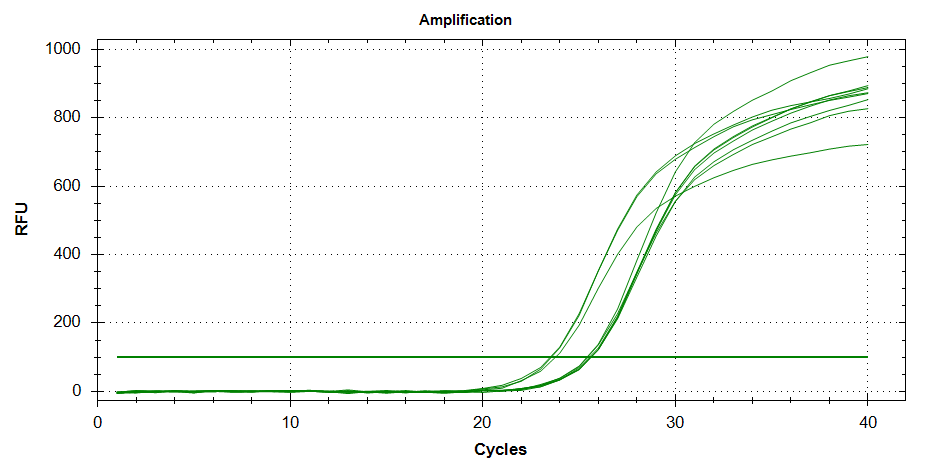

Supplement: Supplementary file 2 [file DataSheet1.ZIP › Raw data/Raw data of PCR/Amplification curves/AC007032.1.png]

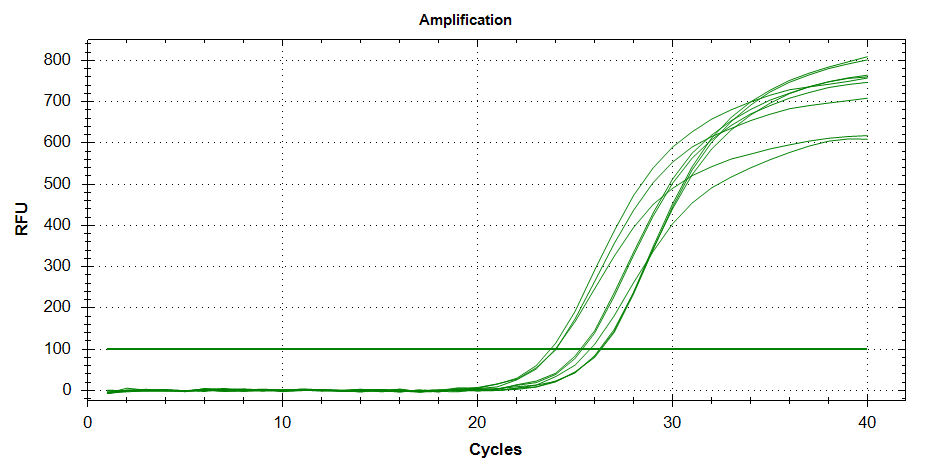

Supplement: Supplementary file 2 [file DataSheet1.ZIP › Raw data/Raw data of PCR/Amplification curves/AC016737.2.png]

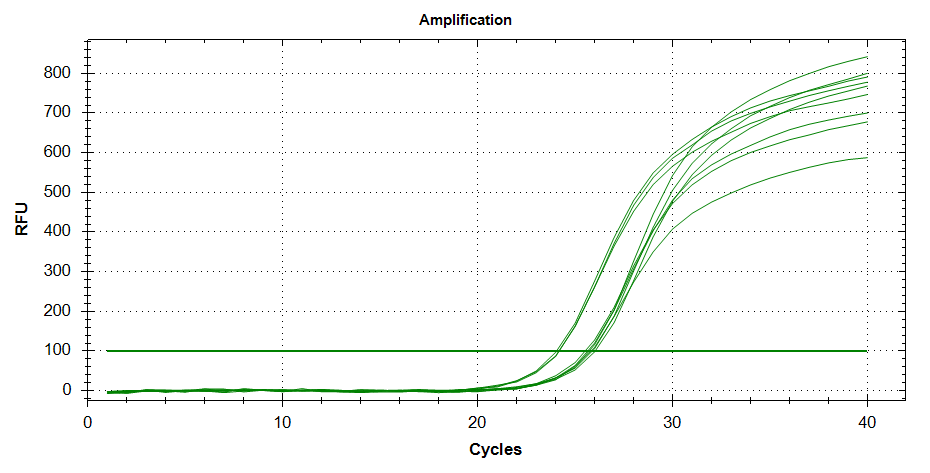

Supplement: Supplementary file 2 [file DataSheet1.ZIP › Raw data/Raw data of PCR/Amplification curves/AL021026.1.png]

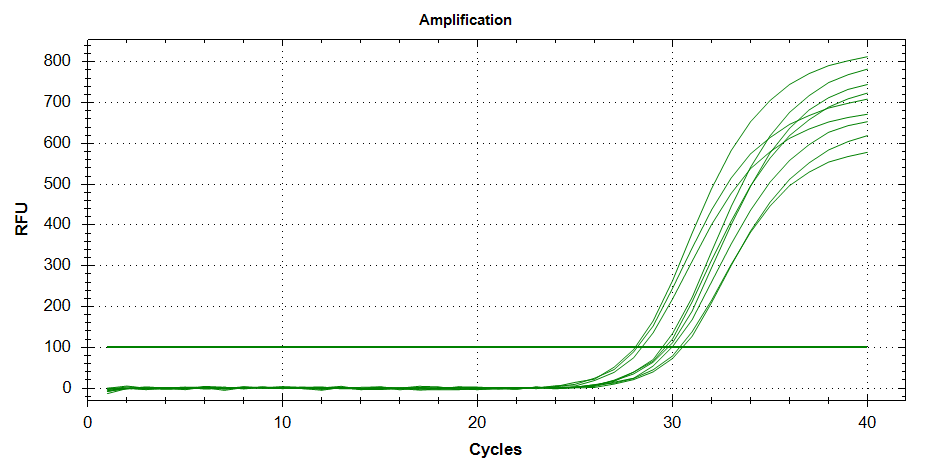

Supplement: Supplementary file 2 [file DataSheet1.ZIP › Raw data/Raw data of PCR/Amplification curves/AL390755.1.png]

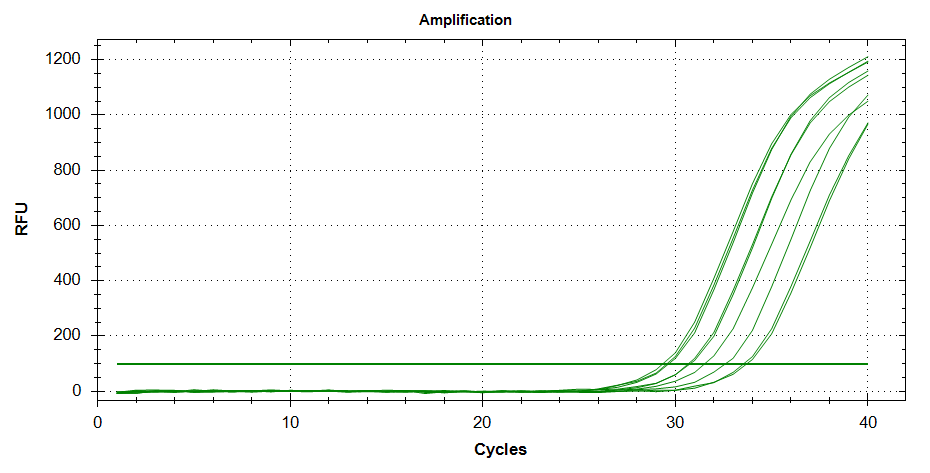

Supplement: Supplementary file 2 [file DataSheet1.ZIP › Raw data/Raw data of PCR/Amplification curves/AL589986.2.png]

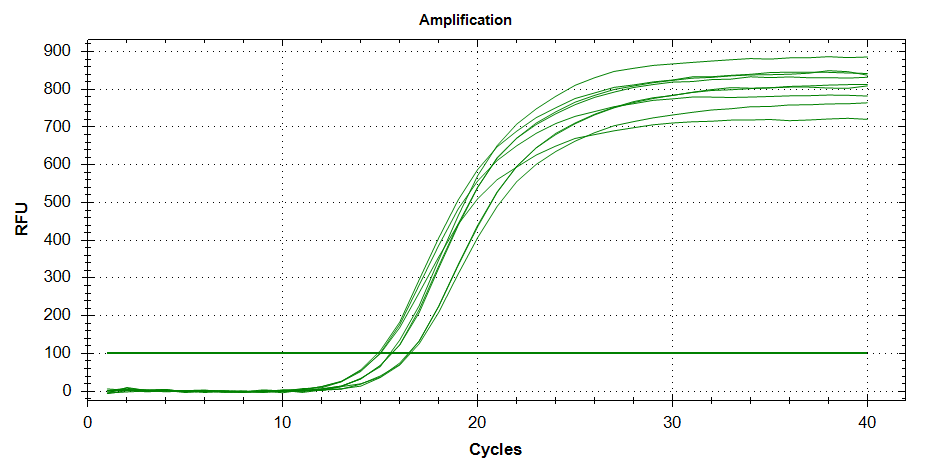

Supplement: Supplementary file 2 [file DataSheet1.ZIP › Raw data/Raw data of PCR/Amplification curves/GAPDH.png]

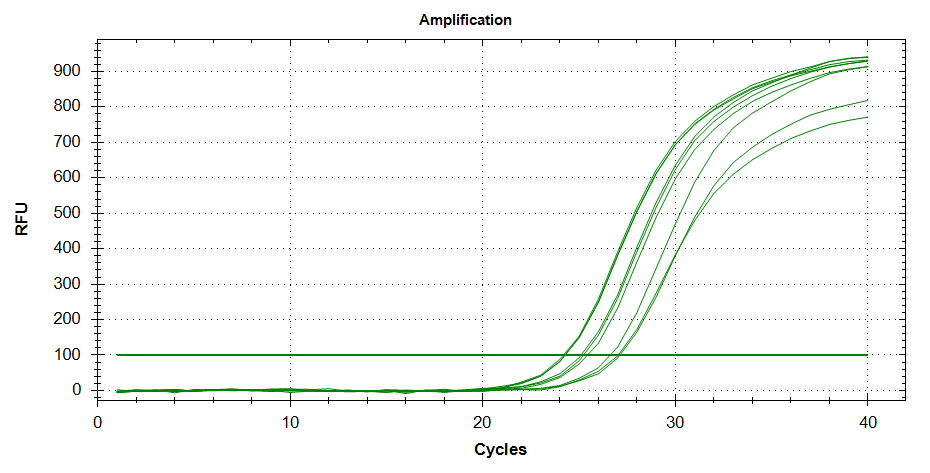

Supplement: Supplementary file 2 [file DataSheet1.ZIP › Raw data/Raw data of PCR/Amplification curves/GAS6-DT.png]

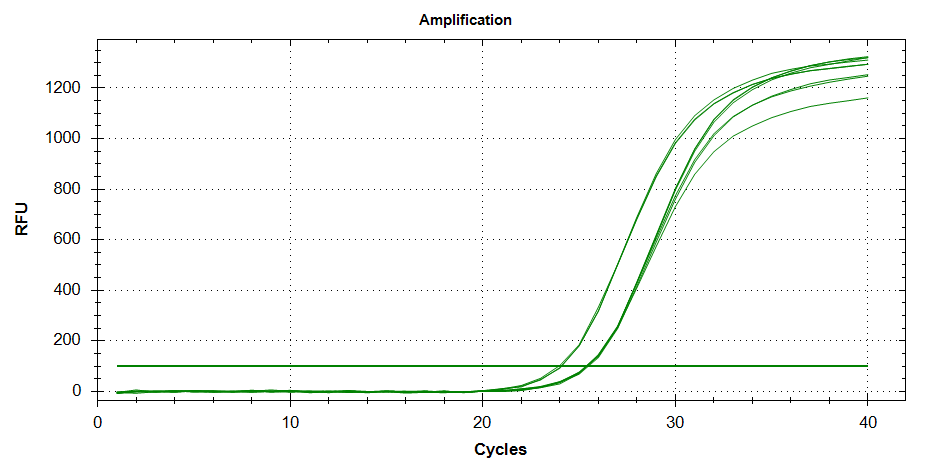

Supplement: Supplementary file 2 [file DataSheet1.ZIP › Raw data/Raw data of PCR/Amplification curves/LINC01281.png]

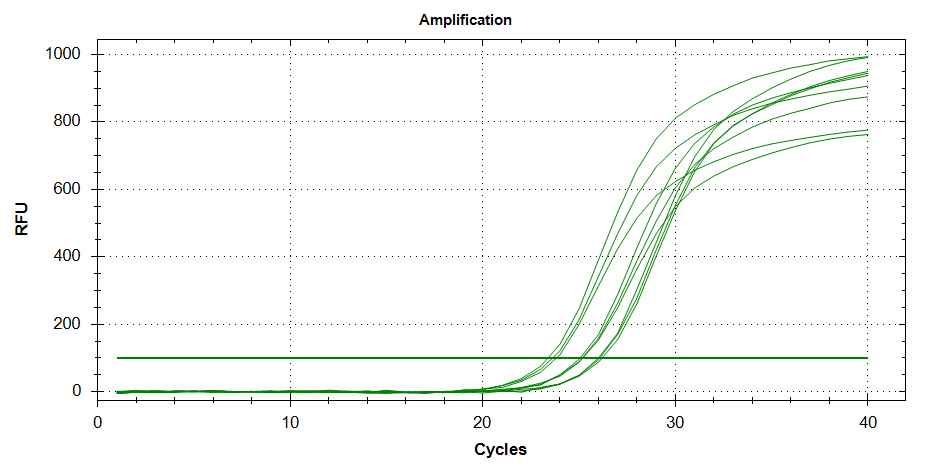

Supplement: Supplementary file 2 [file DataSheet1.ZIP › Raw data/Raw data of PCR/Amplification curves/LINC02198.png]

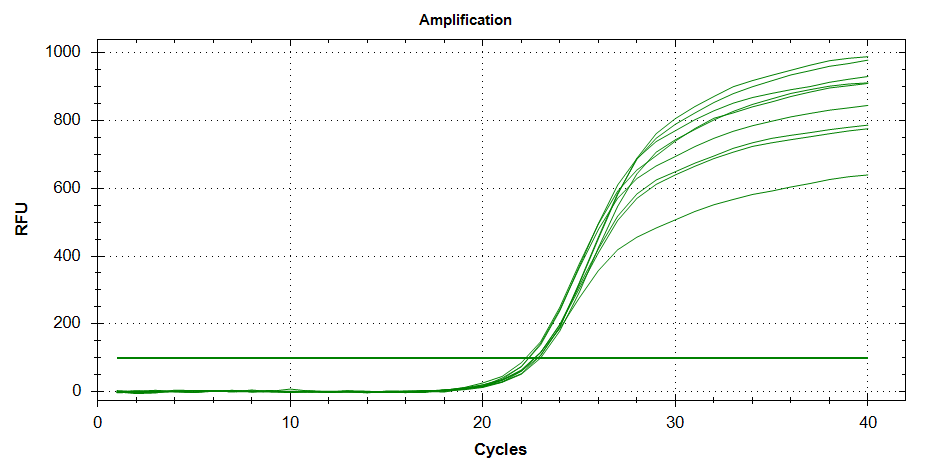

Supplement: Supplementary file 2 [file DataSheet1.ZIP › Raw data/Raw data of PCR/Amplification curves/TMPO-AS1.png]

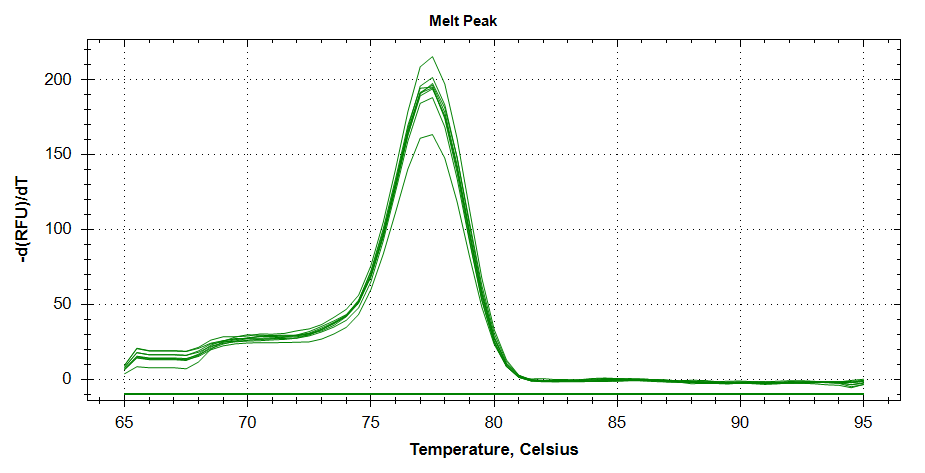

Supplement: Supplementary file 2 [file DataSheet1.ZIP › Raw data/Raw data of PCR/Melting Curve/AC007032.1.png]

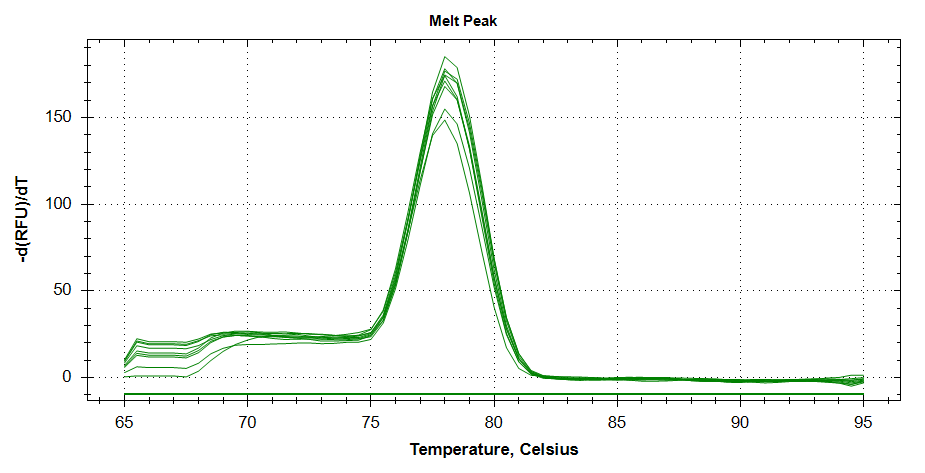

Supplement: Supplementary file 2 [file DataSheet1.ZIP › Raw data/Raw data of PCR/Melting Curve/AC016737.2.png]

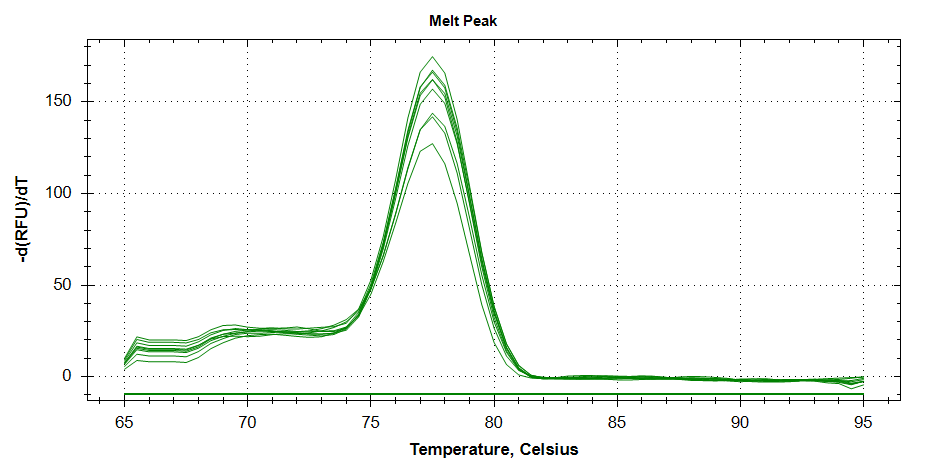

Supplement: Supplementary file 2 [file DataSheet1.ZIP › Raw data/Raw data of PCR/Melting Curve/AL021026.1.png]

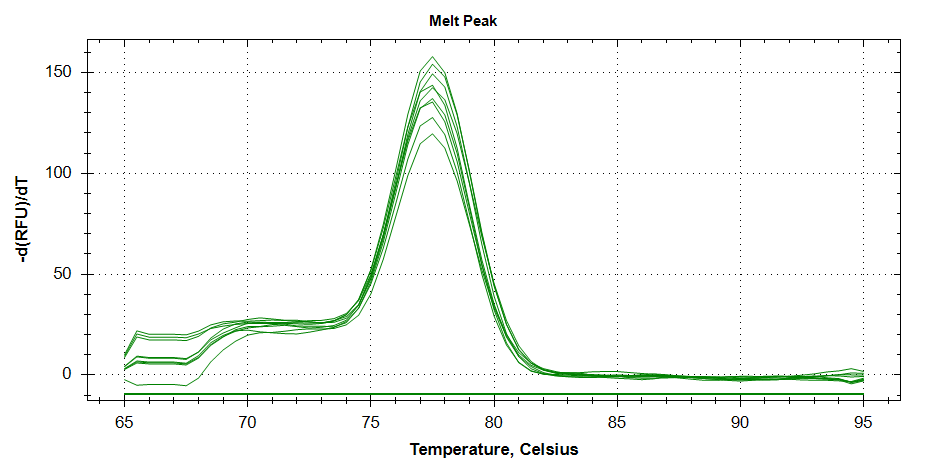

Supplement: Supplementary file 2 [file DataSheet1.ZIP › Raw data/Raw data of PCR/Melting Curve/AL390755.1.png]

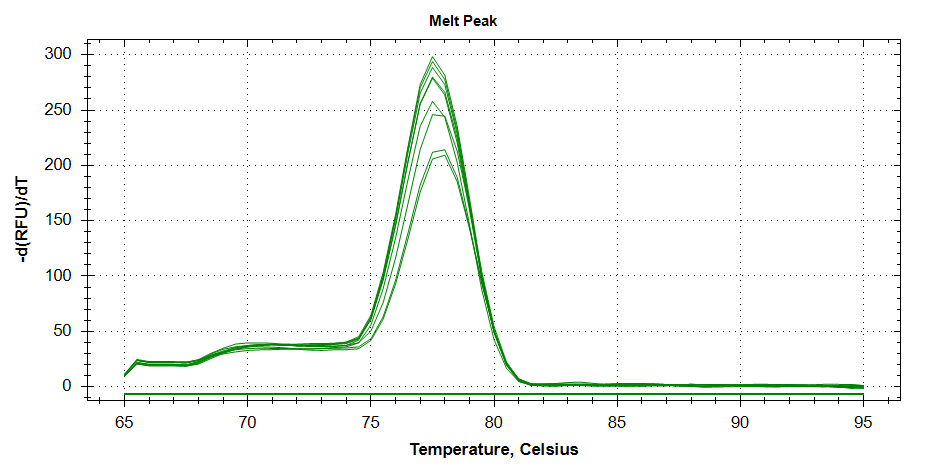

Supplement: Supplementary file 2 [file DataSheet1.ZIP › Raw data/Raw data of PCR/Melting Curve/AL589986.2.png]

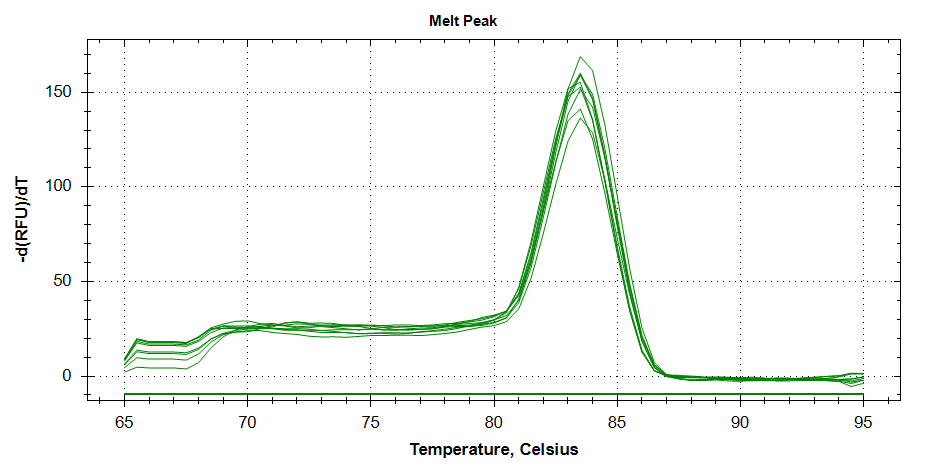

Supplement: Supplementary file 2 [file DataSheet1.ZIP › Raw data/Raw data of PCR/Melting Curve/GAPDH.png]

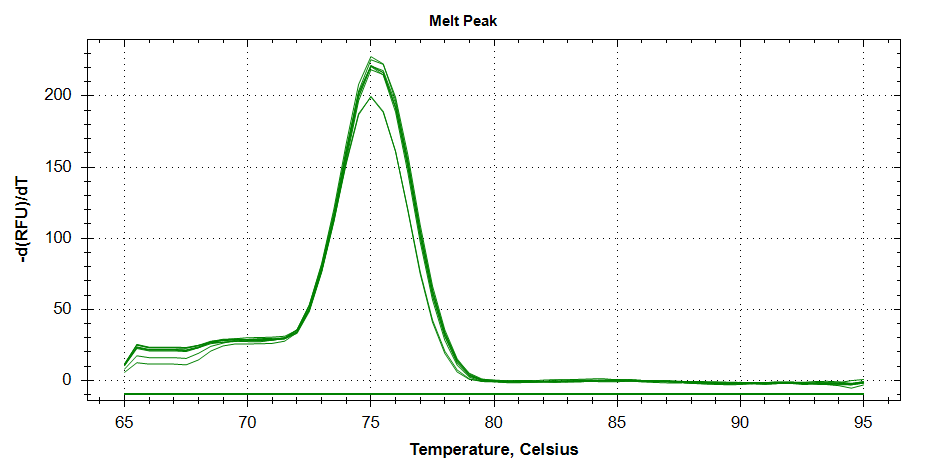

Supplement: Supplementary file 2 [file DataSheet1.ZIP › Raw data/Raw data of PCR/Melting Curve/GAS6-DT.png]

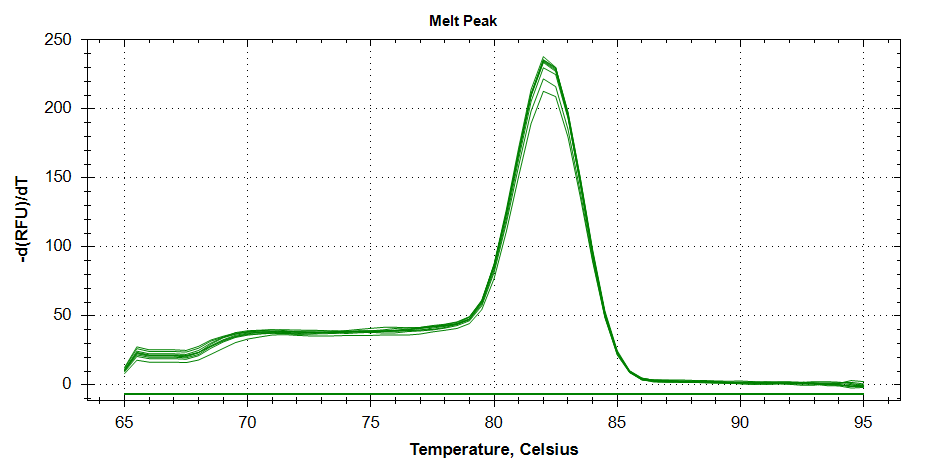

Supplement: Supplementary file 2 [file DataSheet1.ZIP › Raw data/Raw data of PCR/Melting Curve/LINC01281.png]

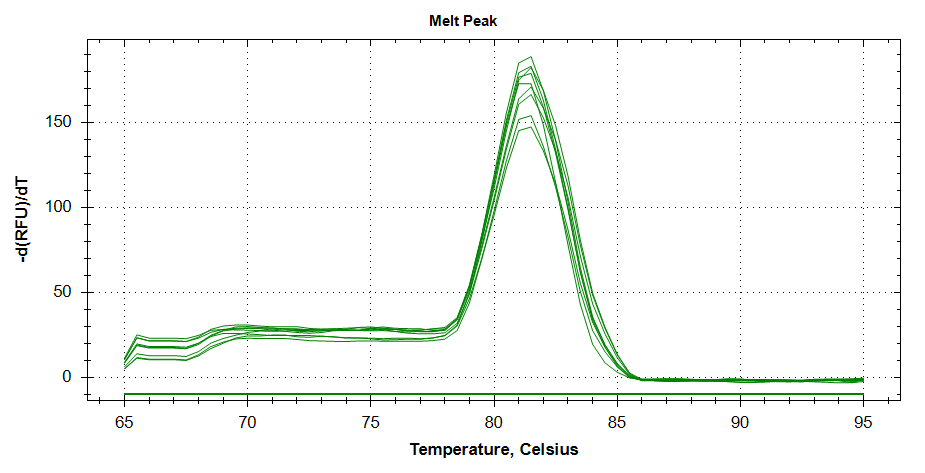

Supplement: Supplementary file 2 [file DataSheet1.ZIP › Raw data/Raw data of PCR/Melting Curve/LINC02198.png]

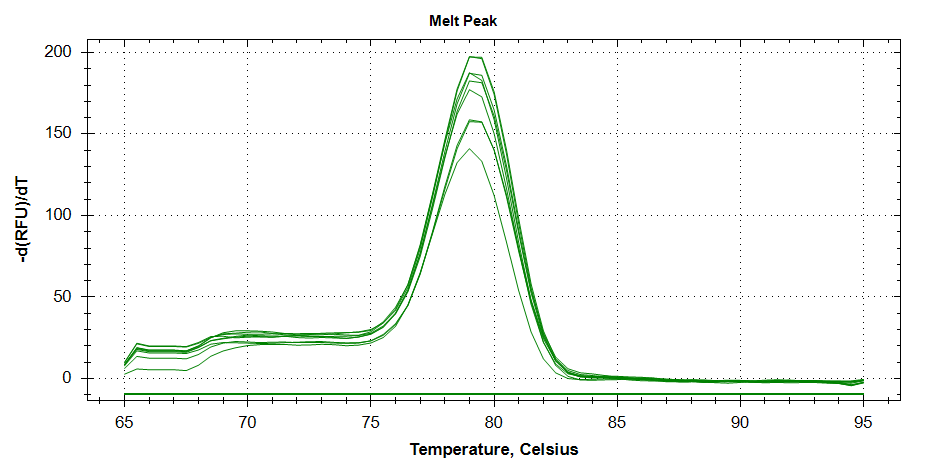

Supplement: Supplementary file 2 [file DataSheet1.ZIP › Raw data/Raw data of PCR/Melting Curve/TMPO-AS1.png]

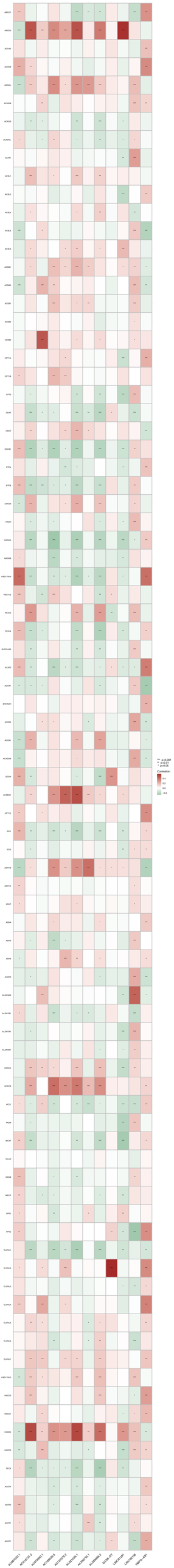

Supplement: Supplementary file 3 [file DataSheet2.ZIP › ▓╣│Σ▓─┴╧/Supplementary Figures 1.pdf]

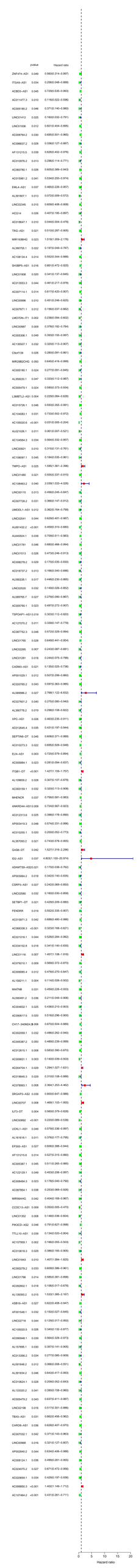

Supplement: Supplementary file 3 [file DataSheet2.ZIP › ▓╣│Σ▓─┴╧/Supplementary Figures 2.pdf]
